# In Situ Exsolution‐Prepared Solid‐Solution‐Type Sulfides with Intracrystal Polarization for Efficient and Selective Absorption of Low‐Frequency Electromagnetic Wave

**DOI:** 10.1002/advs.202403723

**Published:** 2024-07-16

**Authors:** Xiaojun Zeng, Tianli Nie, Chao Zhao, Yanfeng Gao, Xiaofang Liu

**Affiliations:** ^1^ School of Materials Science and Engineering Jingdezhen Ceramic University Jingdezhen 333403 China; ^2^ School of Materials Science and Engineering Shanghai University Shanghai 200444 China; ^3^ School of Materials Science and Engineering Beihang University Beijing 100083 China

**Keywords:** electromagnetic wave absorption, in situ exsolution, intracrystal polarization, low‐frequency, solid‐solution‐type sulfides

## Abstract

The excellent dielectric properties and tunable structural design of metal sulfides have attracted considerable interest in realizing electromagnetic wave (EMW) absorption. However, compared with traditional monometallic and bimetallic sulfides that are extensively studied, the unique physical characteristics of solid‐solution‐type sulfides in response to EMW have not been revealed yet. Herein, a unique method for preparing high‐purity solid‐solution‐type sulfides is proposed based on solid‐phase in situ exsolution of different metal ions from hybrid precursors. Utilizing CoAl‐LDH/MIL‐88A composite as a precursor, Fe_0.8_Co_0.2_S single‐phase nanoparticles are uniformly in situ formed on an amorphous substrate (denoted as CoAl), forming CoAl/Fe_0.8_Co_0.2_S heterostructure. Combing with density functional theory (DFT) calculations and wave absorption simulations, it is revealed that Fe_0.8_Co_0.2_S solid solution has stronger intracrystal polarization and electronic conductivity than traditional monometallic and bimetallic sulfides, which lead to higher dielectric properties in EM field. Therefore, CoAl/Fe_0.8_Co_0.2_S heterostructure exhibits significantly enhanced EMW absorption ability in the low‐frequency region (2–6 GHz) and can achieve frequency screening by selectively absorbing EMW of specific frequency. This work not only provides a unique method for preparing high‐purity solid‐solution‐type sulfides but also fundamentally reveals the physical essence of their excellent EMW absorption performance.

## Graphical abstract

In situ exsolution strategy is developed to construct CoAl/Fe_0.8_Co_0.2_S heterostructures, in which solid‐solution‐type sulfides inherit internal crystal polarization and outstanding dielectric loss ability.

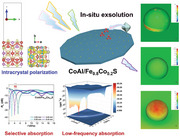

## 1 Introduction

In modern society, electromagnetic waves (EMWs) are ubiquitous and have become an indispensable element in daily life. EMWs have been widely used in the fields of communication, medical, scientific research, military, and so on.^[^ ^1^, ^2^, ^3^ ^]^ For example, the popularity of 5G communication (mainly concentrated in frequencies of 2–6 GHz) has greatly promoted the flourishing development of electronic devices.^[^ ^4^, ^5^, ^6^ ^]^ However, the derived electromagnetic radiation poses a risk to human health,^[^ ^7^, ^8^, ^9^ ^]^ and can seriously interfere with the normal operation of electronic equipment.^[^ ^10^, ^11^, ^12^, ^13^ ^]^ Moreover, in the low‐frequency region, where a large number of electromagnetic waves from various electronic devices are crowded, the electromagnetic compatibility issue is more serious.^[^ ^14^ ^]^ Therefore, there is an urgent need for innovative absorbers that can effectively and selectively absorb low‐frequency EMW. However, the absorption frequencies of the current absorbers are mainly concentrated in the X‐band (8–12 GHz) and Ku‐band (12–18 GHz).^[^ ^15^ ^]^

Metal sulfides are promising candidates for high‐performance EMW absorption materials due to their suitable dielectric constant, high dielectric loss, rich composition/morphology, and good chemical stability.^[^ ^16^, ^17^ ^]^ Currently, metal sulfide‐based EMW absorption materials are mainly concentrated on monometallic sulfides,^[^ ^18^ ^]^ bimetallic sulfides,^[^ ^19^ ^]^ and their composites. Their response to EMW mainly depends on the inherent conductivity, defect polarization, and interfacial polarization. However, most of them show strong EMW absorption at a thin thickness in the high‐frequency range above 8 GHz. Recent successes have demonstrated that precisely adjusting the dielectric properties of materials is a feasible way to enhance low‐frequency absorption of EMW.^[^ ^10^, ^20^, ^21^ ^]^ Compared with conventional sulfide absorbers, solid‐solution‐type sulfides can generate abundant intracrystal polarization centers due to the incorporation of different metal ions into the cation lattice positions, which is more conducive to optimizing and regulating dielectric properties.^[^ ^22^, ^23^ ^]^ Therefore, solid‐solution‐type sulfides are expected to be a good candidate for low‐frequency EMW absorption material. However, the significant effect of intracrystalline polarization on EWM losses has not been revealed yet. Relevant research will deepen the understanding of EMW absorption mechanism, and promote the design of new low‐frequency EMW absorption materials.

Currently, sulfide‐based EMW absorption materials are generally prepared by the liquid‐phase method, which easily causes the phase separation of bimetallic sulfides. For example, Song et al. used the coprecipitation method to in situ grow (Co,Fe)‐based sulfides on carbon aerogels to enhance EMW absorption, and finally obtained carbon aerogels loaded with a mixture of monometallic sulfides (Co_x_S_y_ and Fe_x_S_y_).^[^ ^24^ ^]^ In order to obtain high‐purity and stable solid‐solution‐type sulfide phases, the in situ exsolution method based on solid‐state reactions provides an effective solution strategy.^[^ ^25^ ^]^ Under high‐temperature conditions, multiple metal atoms in the matrix can be extruded from crystal lattice together, and quickly combine with nonmetal atoms on the matrix surface due to their high activity, thereby avoiding phase separation. Moreover, the nanoparticles formed by the in situ exsolution route are small in size and are highly dispersed on the matrix, which facilitates enhancing the interface polarization and stability.^[^ ^26^, ^27^ ^]^

The selection of an appropriate matrix is crucial for the in situ exsolution method. Due to the rapid in situ exsolution process at high temperatures, multiple metal ions in the same matrix will rapidly precipitate from the lattice and combine with nonmetallic atoms to form various metal compounds, leading to phase separation. To solve this problem, it is necessary to maintain a certain distance between metal ions to avoid phase separation caused by excessively fast reaction rates. Therefore, choosing two matrices to supply different metal ions and promote their reaction with extra nonmetallic atoms at the interface to form bimetallic compounds is a feasible strategy. 2D layered bimetallic hydroxide CoAl‐LDH with high aspect ration and numerous metal cations in an unsaturated coordination state on its surface, providing more sites for in situ exsolution and directional epitaxial growth.^[^ ^28^ ^]^ Moreover, metal–organic frameworks (MOFs) with abundant metal units can also provide highly active and well‐dispersed metal reaction sites through in situ exsolution pathways, making them potential candidates for supplying the second type of metal atom. Due to the similar competitive ability of metal Fe and metal Co in CoAl‐LDH toward S^2−^, a Fe‐based MOF (MIL‐88A) is selected as the transition metal source.^[^ ^29^ ^]^ In this work, we choose CoAl‐LDH/MIL‐88A composite as the matrix and use in situ exsolution method to prepare high‐purity Fe_0.8_Co_0.2_S solid solution, which is loaded on LDH‐derived amorphous sheets. Fe_0.8_Co_0.2_S possesses strong intracrystal polarization and forms numerous interfaces with the matrix. Therefore, the CoAl‐Fe_0.8_Co_0.2_S heterostructure exhibits currently the best EMW absorption performance in the low‐frequency band (2–6 GHz). At a matching thickness of 3.52 mm, the reflection loss (*R*_L_) reaches ─65.26 dB at 6.0 GHz. Moreover, CoAl/Fe_0.8_Co_0.2_S also exhibits attractive frequency selection behavior in the low‐frequency range. When the material thickness changes from 3.95, 5.0, 6.5 to 8.45 mm, the sharp absorption peak can effectively absorb undesirable EM waves of specific frequency. The importance of intracrystal polarization on low‐frequency absorption is revealed combing with first principles calculation.

## 2 Results and Discussion

**Figure 1:**
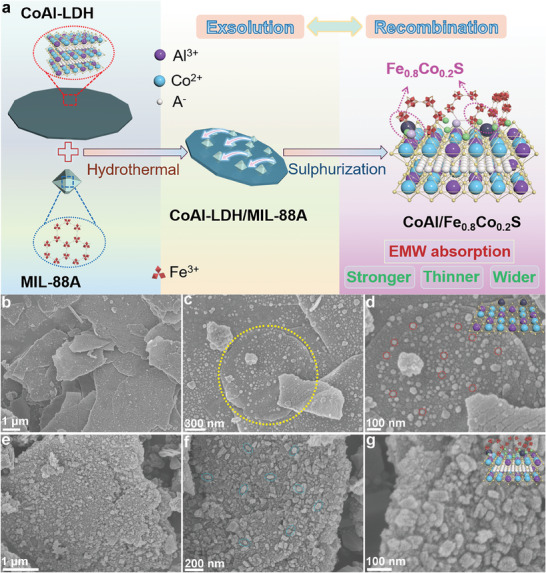
a) Scheme showing the formation process of CoAl/Fe_0.8_Co_0.2_S heterostructure. SEM images of b–d) CoAl/Co_1‐x_S and e–g) CoAl/Fe_0.8_Co_0.2_S heterostructure.

The synthesis process of CoAl/Fe_0.8_Co_0.2_S is visually presented in **Figure** **1**a. Initially, a well‐defined ultrathin nanosheet structure of CoAl‐LDH was fabricated through a co‐precipitation method. This layered CoAl‐LDH, characterized by its distinctive 2D nature and substantial specific surface area, boasts an abundance of unsaturated metal ions. This augmented availability of in situ nucleation sites is pivotal to the subsequent stages. Subsequently, MIL‐88A was meticulously grown upon the CoAl‐LDH scaffold. The morphology of MIL‐88A, characterized by its spindle‐like configuration and synthesized through the linkage of Fe^3+^ to oxygen atoms present in FA, maintains a consistent and smooth contour.^[^ ^30^ ^]^ The prolific nucleation sites inherent in CoAl‐LDH harmoniously interact with iron ions in MIL‐88A, facilitating comprehensive coordination between the two entities. Finally, CoAl‐LDH/MIL‐88A composite underwent sulfide heat treatment, culminating in the formation of a 3D construct of CoAl/Fe_0.8_Co_0.2_S heterostructure. This synthetic material exhibits a copious array of interfaces, engendering a rich structural complexity. The presence of organic components imparts escalating reducibility as heat treatment progresses, thus instigating the impetus for exsolution. The interaction dynamics during the heat treatment process are a compelling sequence. Fe^3+^ experiences partial exsolution in response to reducing conditions, subsequently undergoing reduction to Fe^2+^ through interaction with graphitic carbon. Ultimately, a union of Co^2+^, Fe^2+^, and S^2−^ orchestrates the formation of the Fe_0.8_Co_0.2_S solid solution.^[^ ^31^ ^]^ Notably, the synchronous in situ exsolution of Co^2+^ and Fe^2+^ promotes the steadfast adherence of solid‐solution‐type sulfides upon the material surface. This intrinsic firmness curtails the possibility of agglomeration, thereby enhancing material stability. Meanwhile, the bimetallic nanoparticles conspicuously amplify the interfacial diversity within the material.

The microstructure of the samples was meticulously investigated using field emission scanning electron microscopy (FESEM). As portrayed in Figure S1 (Supporting Information), CoAl‐LDH is an ultrathin nanosheet structure with a smooth surface. Obviously, the CoAl/Co_1‐x_S retains its ultrathin nanosheet structure (≈1.5 µm in diameter and ≈80 nm in thickness).^[^ ^28^ ^]^ Moreover, after the sulphurization process, a larger number of small uniform nanoparticles are derived from the surface of the nanosheets, as illustrated in Figure 1b–d and Figure S2 (Supporting Information). This phenomenon is attributable to the creation of reduction conditions generated during the pyrolysis of thiourea, facilitating the exsolution of Co^2+^ from its original position. Consequently, Co^2+^ reacts with S^2−^ to generate metal sulfide (Co_1‐x_S) nanoparticles. The achieved Co_1‐x_S nanoparticles with a diameter of ≈25 nm can be observable in their uniform and regular size. Figure S3 (Supporting Information) shows the SEM image of MIL‐88A, which shows a large number of uniform spindles with a length of ≈350 nm. The morphology of the CoAl/Fe_0.8_Co_0.2_S heterostructure is depicted in Figure 1e–g. Figure 1e exhibits that there are numerous nanoparticles attached to the surface of CoAl/Co_1‐x_S. These crystal‐like nanoparticles are Fe_0.8_Co_0.2_S solid solutions, consistent with the above conclusions. Meanwhile, the uneven and relatively larger nanoparticles on the surface are attributed to the recrystallization from the recombination process. Apparently, the recombination process promotes the tight binding and chemical interaction between NC/FeS and CoAl/Co_1‐x_S, which further promotes interface polarization. Notably, the CoAl/Co_1‐x_S nanosheets exhibit a propensity to thicken (Figure 1f). This phenomenon is attributed to the uniform and dense nature of Fe_0.8_Co_0.2_S nanoparticles formed through recombination of exsolved Co^2+^ and Fe^3+^, resulting in comprehensive coverage of the CoAl/Co_1‐x_S surface. The intricate composition of the heterostructure is further underscored in Figure 1g, revealing an abundance of heterogeneous interfaces within the material. These multiple heterogeneous interfaces endow the material with an exceptional potential for interfacial polarization. Figure S4 (Supporting Information) presents the energy‐dispersive X‐ray Spectroscopy (EDS) elemental mapping images of CoAl/Fe_0.8_Co_0.2_S. From the distribution patterns, it is evident that Al, Fe, Co, S, C, N, and O elements exhibit a uniform distribution over the CoAl/Fe_0.8_Co_0.2_S heterostructure. Additionally, the elemental strength of Co and N is relatively weak, which is attributed to their relatively low content, which is consistent with the above results.

**Figure 2:**
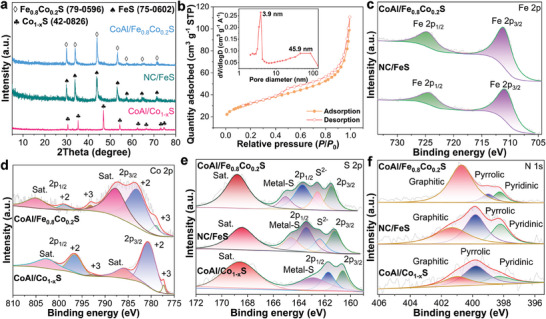
a) XRD patterns of CoAl/Co_1‐x_S, NC/FeS, and CoAl/Fe_0.8_Co_0.2_S heterostructure. b) N_2_ adsorption‐desorption isotherms of CoAl/Fe_0.8_Co_0.2_S. High‐resolution XPS spectra of c) Fe 2p, d) Co 2p, e) S 2p, and f) N 1s for the samples.

The crystalline composition of the CoAl/Fe_0.8_Co_0.2_S heterostructure was investigated through X‐ray diffraction (XRD). The synthesized CoAl‐LDH shows diffraction peaks at 23.7, 34.7, 39.5, and 47.0 (Figure S5, Supporting Information), which are consistent with the (006), (012), (015), and (018) crystal planes of the CoAl‐LDH phase,^[^ ^28^ ^]^ indicating its high purity. Notably, the conversion of CoAl‐LDH into a singular Co_1‐x_S (PDF #42‐0826) crystalline phase ensued subsequent to thiourea‐induced heat treatment (**Figure** **2**a). Furthermore, the reductive environment engendered exclusively facilitated the in situ exsolution of transition metal Co^2+^, ultimately forming cobalt sulfide. The prepared MIL‐88A exhibits diffraction peaks corresponding to the standard card of MIL‐88A,^[^ ^32^ ^]^ as depicted in Figure S6 (Supporting Information). During the sulphurization process, the intrinsic organic constituents within MIL‐88A augmented the reducing properties, thereby prompting the detachment of Fe^3+^ from its original position, particularly from the oxygen element within FA. Consequently, Fe^3+^ gradually reduces to Fe^2+^, subsequently desolvating to the surface of the material and engendering the crystalline phase of FeS (PDF #75‐0602). Evidently, both CoAl‐LDH and MIL‐88A post‐thiourea induction exhibited a solitary crystalline phase, demonstrating the efficacy of the exsolution process. Moreover, after sulphurization, the CoAl‐LDH/MIL‐88A composite shows a phase of Fe_0.8_Co_0.2_S solid solution (PDF #42‐0826). This intriguing finding, as discussed earlier, emphasizes the role of abundant nucleation sites on CoAl‐LDH, promoting a closer interaction with MIL‐88A. The absence of a Co_1‐x_S phase after sulphurization signifies the exclusive propensity of exsolved Co^2+^ to unite with Fe^2+^ and S^2−^ to achieve Fe_0.8_Co_0.2_S, indicating the complete amalgamation of exsolved Co^2+^ within CoAl‐LDH. However, pure CoFe‐LDH undergoes phase separation after sulphurization (see details in the Supporting Information), forming FeCoS_2_, Fe_7_S_8_, and Co_9_S_8_ phases (Figure S7, Supporting Information), which fully demonstrates the importance of CoAl‐LDH and MIL‐88A in promoting the formation of solid‐solution‐type sulfides. Except for the diffraction peaks of Co_1‐x_S, FeS, and Fe_0.8_Co_0.2_S phases, no additional peaks can be detected in the three XRD patterns, which indicates that the original CoFe‐LDH phase converted to an amphous phase (denoted as CoAl). Additionally, the surface area and porous architecture were extensively examined using N_2_ adsorption‐desorption isotherms, as shown in Figure 2b. Importantly, CoAl/Fe_0.8_Co_0.2_S generates an elevated surface area of 112.7 m^2^ g^−1^. The corresponding pore size distribution includes mesopores (3.9 nm) and macropores (45.9 nm). This intricate pore hierarchy contributes to the presence of a multilevel pore structure within CoAl/Fe_0.8_Co_0.2_S, which is a pivotal attribute fostering optimal conditions for EMW absorption.^[^ ^33^ ^]^

To investigate the elemental binding configurations and valence states, the samples underwent comprehensive X‐ray photoelectron spectroscopy (XPS) analysis. The XPS survey spectra in Figures S8–10 (Supporting Information) confirm the successful sulphurization of CoAl‐LDH, MIL‐88A, and CoAl‐LDH/MIL‐88A. A closer examination of these spectra reveals the presence of five elements within the CoAl/Co_1‐x_S sample, with the highest abundance being attributed to O (Figure S8, Supporting Information). In contrast, CoAl/Fe_0.8_Co_0.2_S encompasses a total of seven elements (Figure S10, Supporting Information), showcasing a heightened concentration of Fe originating from the incorporation of MIL‐88A. The augmented C content in CoAl/Fe_0.8_Co_0.2_S also indicates that the introduction of MOF material contributes to enhancing the reduction capabilities of the material. Moreover, the atomic ratio of Co:Fe is 5:2, which is basically consistent with the XRD result. The high‐resolution XPS spectra of Fe 2p in NC/FeS and CoAl/Fe_0.8_Co_0.2_S can encompass two convolution peaks (Figure 2c), namely Fe 2P_3/2_ and Fe 2p_1/2_.^[^ ^34^ ^]^ A discerning focus on the high‐resolution XPS spectra of Co 2p for CoAl/Co_1‐x_S and CoAl/Fe_0.8_Co_0.2_S uncovers the coexistence of +2 and +3 peaks within the Co 2p convolution profile (Figure 2d), accompanied by two satellite peaks. The absence of distinct metallic Co peaks affirms the direct binding of exsolved Co^2+^ in the formation of metal sulfide nanoparticles, devoid of further reduction.^[^ ^35^ ^]^ Importantly, the formation of solid‐solution‐type sulfides leads to a positive shift in the binding energy of Co 2p_3/2_ and Co 2p_1/2_, which is attributed to the electronic interaction between Co and Fe.^[^ ^36^ ^]^ On the other hand, the convolution peaks in the high‐resolution XPS spectra of S 2p for CoAl/Co_1‐x_S, NC/FeS, and CoAl/Fe_0.8_Co_0.2_S reveal the presence of S 2p_3/2_, S 2p_1/2_, Metal‐S, and a satellite peak (Figure 2e). Interestingly, the Metal‐S peak aligns with the characteristics of CoAl/Co_1‐x_S, as evidenced by XRD. In contrast, the convoluted peaks after vulcanization of MIL‐88A and the complex include S 2p_3/2_, S 2p_1/2_, S^2−^ and M─S, accompanied by a satellite peak.^[^ ^37^ ^]^ Similarly, the Metal‐S peak in NC/FeS should be FeS, whereas the Metal‐S peak after vulcanization of complex is a coexistence of solid‐solution‐type sulfides (Fe_0.8_Co_0.2_S) and FeS, along with an additional S^2−^ peak relative to Co_1‐x_S.^[^ ^38^ ^]^ Furthermore, the N 1s convolution peaks of CoAl/Co_1‐x_S, NC/FeS, and CoAl/Fe_0.8_Co_0.2_S can be dissected into pyridine N, pyrrole N, and graphite N components through deconvolution (Figure 2f).^[^ ^39^ ^]^ The introduction of N heteroatoms promotes dipolar polarization, thereby improving the EMW absorption performance. Additionally, the convolution peaks in the high‐resolution XPS spectra of O 1s for CoAl/Co_1‐x_S, NC/FeS, and CoAl/Fe_0.8_Co_0.2_S contain distinct configurations (Figure S11, Supporting Information), including C─O, C = O, and Metal─O.^[^ ^40^ ^]^ Finally, the convolution peaks of C 1s are depicted in Figure S12 (Supporting Information). The fitted three peaks can be assigned to C─C, C─N, and C─O, which further confirms the existence of N heteroatoms.

**Figure 3:**
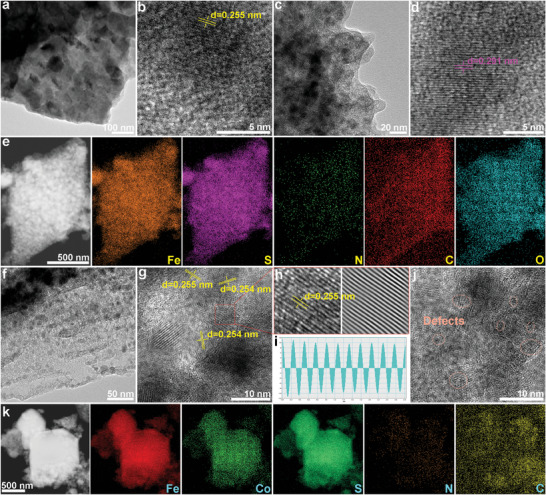
a) TEM and b) HRTEM images of CoAl/Co_1‐x_S. c) TEM and d) HRTEM images of NC/FeS. e) HAADF‐STEM images and corresponding elemental mapping images of NC/FeS. f) TEM images, g,h,j) HRTEM images, and i) lattice interplanar spacings of CoAl/Fe_0.8_Co_0.2_S heterostructure. k) HAADF‐STEM images and corresponding elemental mapping images of CoAl/Fe_0.8_Co_0.2_S.

The internal structure of the sample is subjected to further scrutiny through transmission electron microscopy (TEM), revealing insightful details as shown in **Figure** **3**. Figure 3a and Figure S13 (Supporting Information) vividly illustrate the profusion of Co_1‐x_S nanoparticles thriving on the surface of CoAl/Co_1‐x_S nanosheets. Upon closer magnification, these small nanoparticles unveil a uniform size and densely packed arrangement, a compelling indicator of the pronounced in situ exsolution for Co^2+^. The high‐resolution TEM (HRTEM) image in Figure 3b shows a lattice interplanar spacing of 0.255 nm, which can be assigned to the (101) plane of the Co_1‐x_S phase.^[^ ^41^ ^]^ Turning attention to Figure 3c and Figure S14 (Supporting Information), a larger number of small nanoparticles are embedded in the spindle‐like NC/FeS, which is the result of the dissolution of FeS nanoparticles during the sulphurization process. The lattice interplanar spacing of 0.291 nm in Figure 3d is consistent with the (110) plane of the FeS phase, further proving the success of sulphurization for Fe^2+^. Importantly, high‐angle annular dark field scanning transmission electron microscopy (HAADF‐STEM) images and corresponding EDS elemental mapping images reveal the uniform distribution of Fe, S, N, C, and O in the spindle‐like NC/FeS (Figure 3e). Figure 3f and Figure S15 (Supporting Information) display the TEM image of the CoAl/Fe_0.8_Co_0.2_S heterostructure, which confirms the highly porous nanosheet structure. Meanwhile, some small nanoparticles are distributed around the heterostructure, which is derived from the exsolution and recombination during the sulphurization process. The lattice interplanar spacings of 0.255 and 0.254 nm are clearly visible in Figure 3g–i and Figure S16 (Supporting Information), which can be attributed to the (101) plane of FeCoS_2_.^[^ ^42^ ^]^ This result further confirms the existence of Fe_0.8_Co_0.2_S in the CoAl/Fe_0.8_Co_0.2_S heterostructure, which is consistent with the XRD results. Importantly, CoAl/Fe_0.8_Co_0.2_S heterostructure enables numerous defects, as shown in Figure 3j. These defects can greatly promote dipolar polarization, thereby enhancing the EMW absorption performance. Figure 3k and Figure S17 (Supporting Information) unveil the HAADF‐STEM images and corresponding elemental mapping images of CoAl/Fe_0.8_Co_0.2_S, indicating a uniform elemental distribution (Fe, Co, S, N, C, O, Al) across the surface of the heterostructure. This uniform distribution underscores the normative exsolution of transition metal ions in the material, contributing to a comprehensive regulation of the internal composition and structure for the heterostructure.

**Figure 4:**
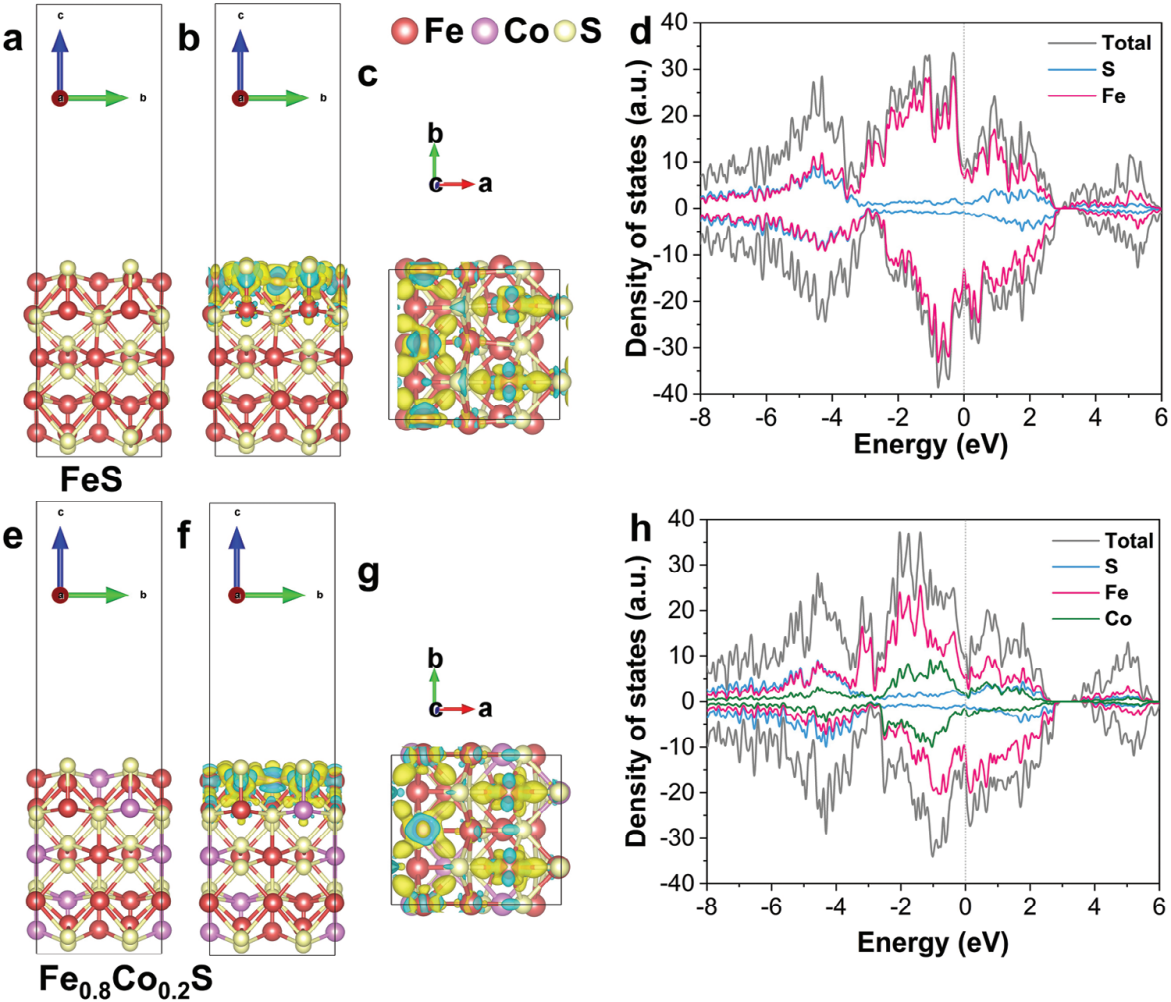
Schematic models of the optimized a) FeS and b) Fe_0.8_Co_0.2_S. The charge density maps of b,c) FeS and f,g) Fe_0.8_Co_0.2_S systems (The level of the isosurface is set to 0.01 e Å^−3^). Density of state of d) FeS and h) Fe_0.8_Co_0.2_S.

To reveal the unique physical advantages of solid‐solution‐type sulfides, we conducted density functional theory (DFT) calculations to compare the charge density and density of states (DOS) of monometallic sulfides (FeS) and solid‐solution‐type sulfides (Fe_0.8_Co_0.2_S). The optimized models are shown in **Figure** **4**a,e, and their charge densities are displayed in Figure 4b,c,f,g. The irregular yellow and blue‐green parts in the charge density map represent the accumulation and dissipation of electrons, respectively. It can be seen that after introducing Co atoms to replace some Fe atoms, the yellow and blue–green parts of the model increase to different degrees, indicating an increase in electron accumulation and dissipation. Moreover, electron dissipation is more pronounced near the position replaced by Co, indicating that the introduction of Co has disrupted the original polarization equilibrium and constructed a new polarization center near Co. Figure 4d,h shows the DOS of FeS and Fe_0.8_Co_0.2_S, respectively. Obviously, no significant bandgap was observed at the Fermi level of FeS and Fe_0.8_Co_0.2_S, indicating that both materials exhibit metallic‐like properties and therefore enable strong conductivity.^[^ ^43^ ^]^ At the Fermi level, the DOS of FeS is mainly contributed by Fe, while the DOS of Fe_0.8_Co_0.2_S is provided by Fe, Co, and S. The combination effect of Fe, Co, and S shows a higher DOS value, indicating more delocalized electrons and higher conductivity.^[^ ^44^ ^]^ Overall, compared with monometallic sulfides, solid‐solution‐type sulfides have stronger polarization within the crystal and higher conductivity, which are beneficial for stronger dielectric properties in high‐frequency EM fields.

**Figure 5:**
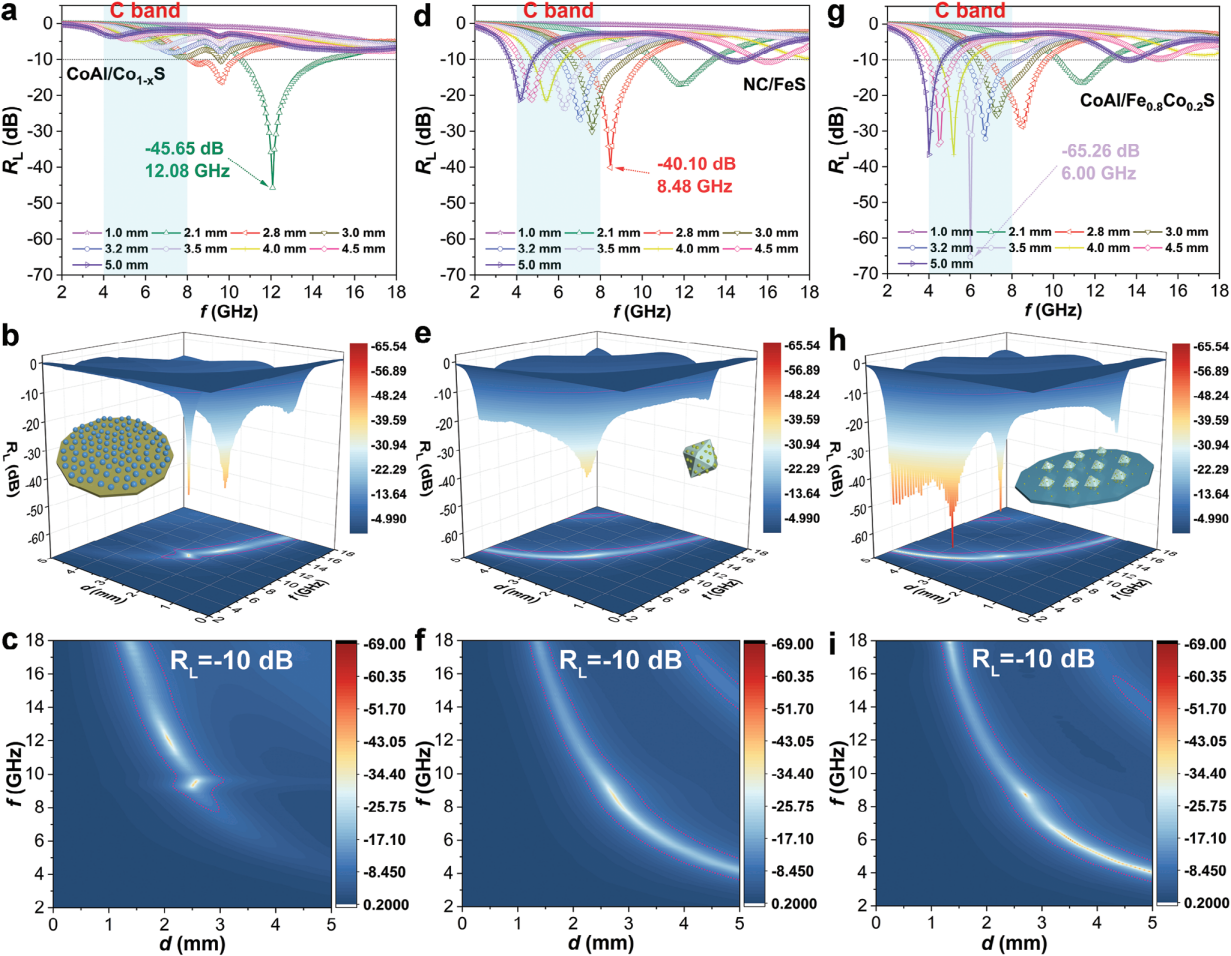
a,d,g) 2D *R*_L_ curves, b,e,h) 3D *R*_L_ curves, and c,f,i) 2D contour maps of a–c) CoAl/Co_1‐x_S, d–f) NC/FeS, and g–i) CoAl/Fe_0.8_Co_0.2_S heterostructure.

To substantiate the EMW absorption capabilities of the heterostructure, comprehensive testing of its electromagnetic parameters was conducted within the frequency range of 2–18 GHz, and the resultant EMW absorption performances are depicted in **Figures** **5** and S18 (Supporting Information). Figure 5a–c illustrates the performance of CoAl/Co_1‐x_S. As proved in our previous work, conventional calcination of CoAl‐LDH generates limited EMW absorption performance due to its single structure,^[^ ^26^ ^]^ which can be attributed to the extremely low permittivity. However, under the influence of thiourea induction, transition metal ions dissolve from CoAl‐LDH and react with S^2−^, leading to the formation of Co_1‐x_S. The highly dispersed metal sulfide nanoparticles on the surface of CoAl species generate rich heterogeneous interfaces, thereby improving dielectric loss. As a consequence, CoAl/Co_1‐x_S demonstrates improved EMW absorption ability with a minimum reflection loss (*R*_L_) value of −45.65 dB at a frequency of 12.08 GHz and an effective absorption bandwidth (EAB) of 4.3 GHz at a matching thickness (*t*) of 2.1 mm. Similarly, the exsolution of Fe^3+^ from the spindle‐shaped MIL‐88A produces a large number of metal sulfide (FeS), which significantly enhances its EMW absorption performance (Figure 5d–f). The *R*_L_ value for NC/FeS is −40.1 dB at a matching thickness of 2.8 mm and a frequency of 8.48 GHz. In comparison, the sulphurization of CoAl‐LDH/MIL‐88A produces more pronounced EMW absorption characteristics (Figure 5g–i). CoAl/Fe_0.8_Co_0.2_S exhibits an *R*_L_ value of −65.26 dB at a matching thickness of 3.5 mm and a frequency of 6.00 GHz, far superior to CoAl/Co_1‐x_S and NC/FeS. Moreover, in the low‐frequency range of 2–6 GHz, the EMW absorption performance of CoAl/Fe_0.8_Co_0.2_S is much better than those of CoAl/Co_1‐x_S and NC/FeS. This means that compared with monometallic sulfides, solid‐solution‐type sulfides have an advantage in improving low‐frequency EMW absorption, which may be attributed to their unique physical properties (explained below). In addition, we adjusted the vulcanization time to optimize EMW absorption, which determines the content of Fe_0.8_Co_0.2_S on the CoAl substrate, thereby regulating the permittivity and permeability of the composite (Figures S18 and S19 (Supporting Information). The CoAl/Fe_0.8_Co_0.2_S heterostructure sulfurized with 10 and 60 min show *R*_L_ values of −17.58 and −58.98 dB, as well as EAB values of 2.5 and 2.72 GHz, respectively. 3D *R*_L_ curves and 2D contour maps of the absorber are employed to visually represent the absorption intensity and bandwidth, as illustrated in Figure 5b,c,e,f,h,i. Compared with CoAl/Co_1‐x_S and NC/FeS, CoAl/Fe_0.8_Co_0.2_S exhibits more prominent EMW absorption ability, especially in the low and middle frequencies.

**Figure 6:**
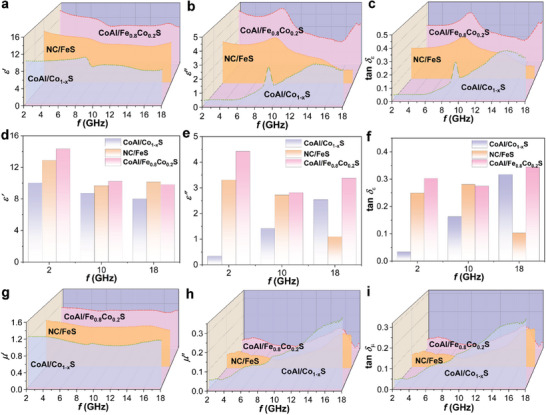
Frequency dependence of a) *ε′*, b) *ε′′*, c) tan *δ*_ε_, g) *µ′*, h) *µ′′*, and (i) tan *δ*_µ_ curves for CoAl/Co_1‐x_S, NC/FeS, and CoAl/Fe_0.8_Co_0.2_S heterostructure. Comparison of d) *ε′*, e) *ε′′*, f) tan *δ*_ε_ values at frequencies of 2, 10, and 18 GHz for the samples.

The electromagnetic parameters of the heterostructures are analyzed based on the complex permittivity (*ε*_r_ = *ε*′ – *jε*″) and complex permeability (*µ*_r_ = *µ*′ – *jµ*″).^[^ ^45^ ^]^ From **Figure** **6**a, it can be seen that the real part of permittivity (*ε*′) for all samples decreases as frequency increases, which is related to the frequency dispersion behavior. The imaginary part of permittivity (*ε*″) for different absorbers is shown in Figure 6b. *ε*″ reflects the EM dissipation capability of the materials. The *ε*″ of CoAl/Fe_0.8_Co_0.2_S follows a similar trend to that of NC/FeS, exhibiting higher *ε*″ in both low and mid‐frequency regions. Compared to CoAl/Co_1‐x_S and NC/FeS, CoAl/Fe_0.8_Co_0.2_S has higher *ε*″, indicating its stronger dielectric dissipation capacity (Figure 6b). The total *ε*″ is composed of the contributions from conduction loss (*ε*″*_σ_*) and polarization loss (*ε*″_p_), and can be expressed as *ε*″  = *ε*″*_σ_* +  *ε*″_p_ = *σ*/(2π*fε*_0_) + 2π*fτ*(*ε*_s_‐*ε*_∞_)/(1+(2π*f*)^2^ *τ* ^2^). According to DFT calculation results, CoAl/Fe_0.8_Co_0.2_S with higher electrical conductivity and higher polarization exhibits larger total *ε*″ and dielectric loss tangent value tan *δ*_ε_ (Figure 6c).^[^ ^46^ ^]^ To further understand the variations of dielectric properties among different materials, a comparison of dielectric parameters and dielectric loss was conducted at frequencies of 2, 10, and 18 GHz, as illustrated in Figure 6d–f. CoAl/Co_1‐x_S shows a gradual decrease in *ε*′ value with increasing frequency, accompanied by an increase in *ε*″ value. This behavior leads to prominent dielectric characteristics of CoAl/Co_1‐x_S in the high‐frequency region. In contrast, both NC/FeS and CoAl/Fe_0.8_Co_0.2_S exhibit substantial dielectric properties at both low and high frequencies, but a significant decrease is observed in the mid‐frequency range, indicating typical dielectric dispersion behavior.

The real part of permeability (*µ*′) for the materials is shown in Figure 6g.^[^ ^47^ ^]^ The *µ*′ values of the three materials have similar variation trends with frequency, indicating that all the samples have magnetic storage capacity.^[^ ^48^ ^]^ Figure 6h displays the imaginary part of permeability (*µ*″) for the materials. CoAl/Co_1‐x_S exhibits an increasing trend in *µ*″ with frequency, indicating more pronounced magnetic loss capabilities in the high‐frequency region. The obvious resonant peaks in the *µ*″ curves of the three samples indicate the presence of natural ferromagnetic resonance at low frequency and exchange resonance at high frequency. The magnetic loss tangent value (tan *δ*_µ_) of CoAl/Fe_0.8_Co_0.2_S is higher than those of CoAl/Co_1‐x_S and NC/FeS, especially at low and medium frequency ranges (Figure 6i), which demonstrates the stronger magnetic loss of solid‐solution‐type sulfides.

**Figure 7:**
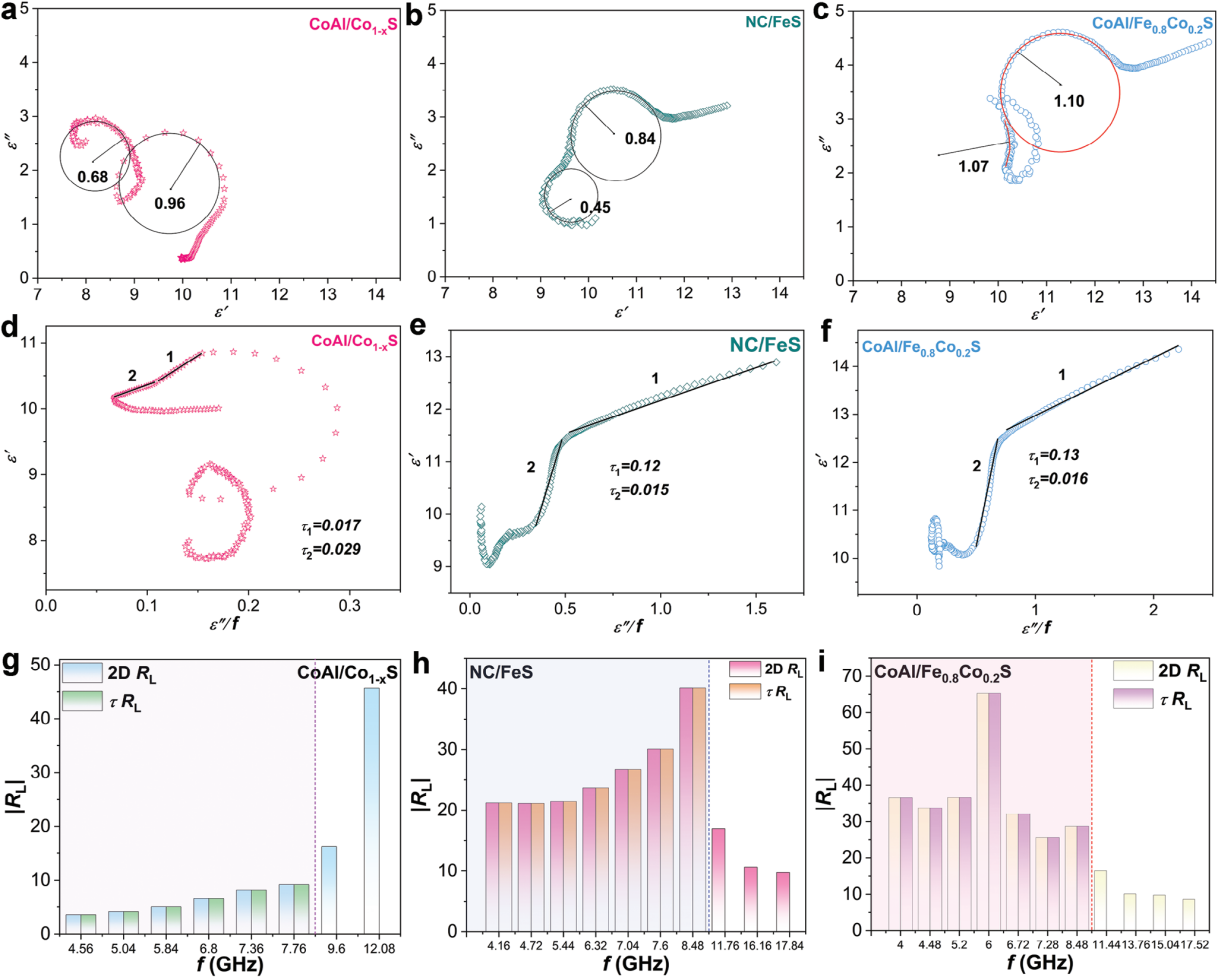
a–c) Cole–Cole and d–f) linear fitting curves of CoAl/Co_1‐x_S, NC/FeS, and c,f) CoAl/Fe_0.8_Co_0.2_S heterostructure. g–i) Comparison of *R*_L_ contained at the frequency corresponding to *τ* and *R*_L_ values contained at a frequency range of 2–18 GHz.

The Debye relaxation phenomenon is an important mechanism for dielectric loss in EMW absorption materials. According to the Debye theory, the relationship between the *ε*′ and the *ε*′′ can be expressed using the following equation:^[^ ^49^ ^]^

(1) $${\left(\varepsilon^{\prime }-\varepsilon_{\infty }\right)}^{2}+\;{\left(\varepsilon^{\prime \prime }\right)}^{2}=\;{\left(\varepsilon_{s}-\varepsilon_{\infty }\right)}^{2}$$

where ε_∞_ represents the dielectric constant at an infinite frequency (high‐frequency limit). *ε*_s_ is the static (or low‐frequency) dielectric constant. Therefore, the *ε′*‐*ε*′′ curve should form a semicircle, (named Cole–Cole semicircle), and one semicircle corresponds to one relaxation process. **Figure** **7** shows the Cole–Cole curves of different materials, which reveals their different polarization relaxation behaviors. All the curves show two distinct semicircles, corresponding to the polarization relaxations from intracrystal polarization and interfacial polarization, respectively.^[^ ^50^ ^]^ The polarization loss (*ε*″_p_) can be reflected by the radii of the Cole–Cole semicircle. As shown in Figure 7a–c, compared with the radii of Cole–Cole semicircles for NC/FeS and CoAl/Co_1‐x_S, CoAl/Fe_0.8_Co_0.2_S presents larger radii of Cole–Cole semicircles, indicating that solid‐solution‐type sulfides have higher polarization relaxation. On the one hand, the stronger intracrystal polarizations of solid‐solution‐type sulfides result in stronger dipole polarization relaxation. On the other hand, the solid‐solution‐type sulfide nanoparticles formed by the in situ exsolution method have smaller sizes and are highly dispersed on the substrate, which generates more heterogeneous interfaces to induce stronger interfacial polarization relaxation. In addition, the presence of an upward‐sloping tail in the *ε′*‐*ε*′′ curves indicates significant conduction loss behavior.^[^ ^51^ ^]^ Compared with NC/FeS and CoAl/Co_1‐x_S, CoAl/Fe_0.8_Co_0.2_S with higher conductivity exhibits a longer tail in a curve, which suggests its higher conduction loss. These results are consistent with the above DFT results that the stronger intracrystal polarization and electronic conductivity of solid‐solution‐type sulfides contribute to their higher polarization loss and conduction loss. The *ε′*‐*ε*′′ curves for CoAl/Fe_0.8_Co_0.2_S with different vulcanization times are shown in Figure S20a,b (Supporting Information). All the metallic sulfides show obvious Cole–Cole semicircles and tails in *ε′*‐*ε*′′ curves, confirming the strong polarization loss and conduction loss. The curve of CoAl/Fe_0.8_Co_0.2_S with a vulcanization time of 30 min has the largest semicircle radii and the longest tail, indicating its strongest dielectric loss.

In the context of dielectric relaxation, the relationship between the *ε*′ and the *ε*′′ is often characterized by a dispersion curve. This curve describes how the polarization processes within the material respond to alternating electric fields applied at different frequencies. When the dielectric loss is related to polarization relaxation, a linear relationship between *ε*″ and *ε*′/*f* can be observed, where *f* represents the frequency of the applied electric field. This type of relationship is typically found in Debye‐like relaxation behavior. The relaxation time (*τ*) characterizes the time scale over which polarization processes occur within the material. Generally, the larger the overall *τ*, the longer the polarization time, reflecting a richer dielectric capacity. *τ* can be calculated from the slope of the linear relationship between *ε*″ and *ε*′/*f* using the following formula:^[^ ^52^ ^]^

(2) τ=1/2πk

where *k* is the slope of the linear function. Not all materials can be fitted linearly because different polarization processes have different polarization times and provide different contributions to the dielectric loss. Figure 7d–f and Figure S20c,d (Supporting Information) represent plots obtained from the fitting of *ε*′ with *ε*″/*f*. CoAl/Co_1‐x_S fits two distinct straight lines which correspond to two relaxation times (*τ*_1_ and *τ*_2_) with values of 0.017 and 0.029, respectively. This indicates the presence of two different relaxation processes in CoAl/Co_1‐x_S. Figure 7e shows the curves obtained from fitting NC/FeS, similarly, two straight lines can be observed. The corresponding relaxation times are *τ*_1_ = 0.12 and *τ*_2_ = 0.015. Furthermore, *τ*_1_ corresponds to a frequency range of 2–5.68 GHz, while *τ*_2_ corresponds to 6.24–9.36 GHz. This indicates that the relaxation processes in the material mainly occur at middle and low frequencies. Figure 7f displays the straight lines obtained from fitting CoAl/Fe_0.8_Co_0.2_S, two distinct lines are also evident. Calculations reveal that the heterostructure achieves *τ*_1_ = 0.13 and *τ*_2_ = 0.016. Compared with CoAl/Co_1‐x_S and NC/FeS, CoAl/Fe_0.8_Co_0.2_S exhibits a longer relaxation time, corresponding to a slower relaxation process, which can be attributed to the stronger intracrystal polarization of solid‐solution‐type sulfides. The frequency range corresponding to *τ*_1_ and *τ*_2_ remains at the low and middle frequencies, implying a significant dielectric loss in this range. Figure 7g–i reveals a comparison between the distribution of *τ* and *R*_L_ values at 2–18 GHz. For these three samples, the frequency range covered by *τ* (*f_τ_*) occupies most of 2–18 GHz, indicating that polarization loss is the main cause of EMW attenuation. However, *f_τ_* of CoAl/Fe_0.8_Co_0.2_S (2–8.48 GHz) is narrower than those of CoAl/Co_1‐x_S (2–8.88 GHz) and NC/FeS (2–9.36 GHz). This phenomenon can be ascribed to the increased conductive loss of CoAl/Fe_0.8_Co_0.2_S.

**Figure 8:**
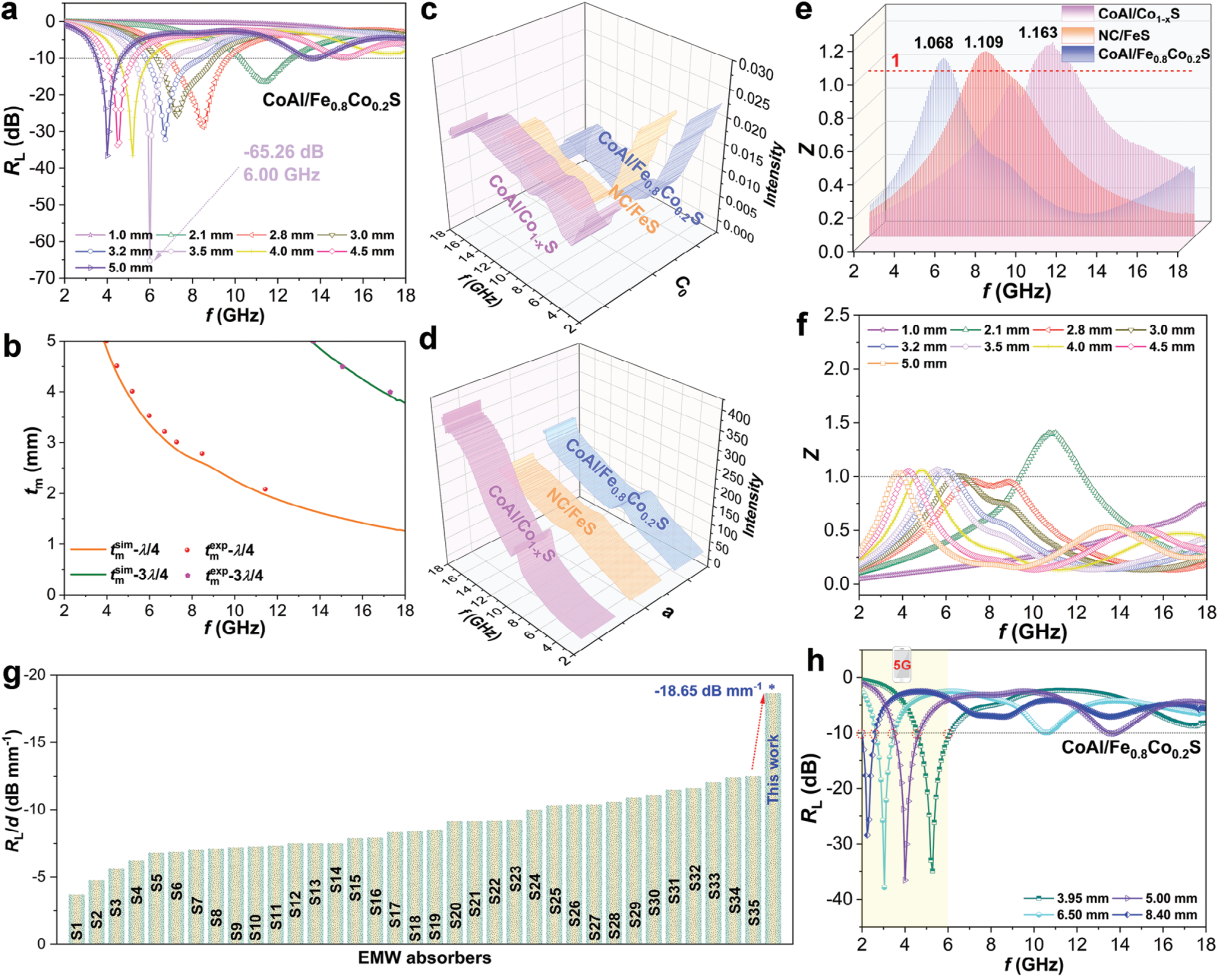
a,b) Simulation of *t*_m_ (*t*_m_^sim^) versus *f*_m_ curves of CoAl/Fe_0.8_Co_0.2_S heterostructure. c) *C*_0_ values of the samples. d) *α* and e) *Z* values of the samples. f) *Z* values of CoAl/Fe_0.8_Co_0.2_S at different thicknesses. g) Comparison of *R*_L_/*d* values for CoAl/Fe_0.8_Co_0.2_S and advanced metal sulfide‐based absorbers in the frequency range of 2–6 GHz (see Table S1 for details). h) 2D *R*_L_ values of CoAl/Fe_0.8_Co_0.2_S in the frequency range of 2–6 GHz.

Magnetic loss is another reason for these samples to absorb EMW. The multiple resonance peaks of *µ*″ demonstrate the coexistence of natural ferromagnetic resonance and exchange resonance. In addition, due to the high conductivity of these samples, an eddy current may be generated under an alternating EM field. Generally, eddy current loss (*C*_0_ = *µ″*(*µ′*)^−2^ *f* ^−1^ = 2π*µ*_0_ *d* ^2^ *ϭ*) can be evaluated by the relationship between *C*_0_ value and frequency. If the *C*_0_ value remains constant with frequency, it indicates that the magnetic loss is primarily due to eddy current loss.^[^ ^53^ ^]^ As shown in **Figure** **8**c, CoAl/Co_1‐x_S displays negligible changes in *C*_0_ values over the entire frequency range, suggesting the significant contribution of eddy current loss. In the case of NC/FeS and CoAl/Fe_0.8_Co_0.2_S, there are more pronounced variations in *C*_0_ values at both low and high frequencies. This implies that the magnetic loss of these two materials involves a combination of natural resonance, exchange resonance, and eddy current loss. The variation trend of *C*_0_ with frequency is similar for CoAl/Fe_0.8_Co_0.2_S with different vulcanization times (Figure S21, Supporting Information).

Theoretically, if the matching thickness (*t*_m_) and matching frequency (*f*_m_) satisfy the quarter‐wavelength matching condition (*t*_m_ = *nc*/(4*f*_m_(|*µ*_r_||*ε*_r_|)^1/2^, *n* = 1,3, 5, …), the reflection loss will reach a maximum. The quarter‐wavelength phase extinction diagram of CoAl/Fe_0.8_Co_0.2_S is shown in Figure 8a,b, and Figure S22 (Supporting Information). The orange and green curves represent the theoretical thicknesses at *λ*/4 and 3*λ*/4, respectively. The experimental thicknesses obtained from testing are represented by solid dots. It is observed that the experimental thicknesses align closely with the theoretical curve, indicating that CoAl/Fe_0.8_Co_0.2_S follows the quarter‐wavelength phase extinction law.

The attenuation constant (*α* = [((2)^1/2^π*f*)/*c*]/{(*µ″ε″* – *µ′ε′*) + [(*µ″ε″* – *µ′ε′*)^2^ + (*ε′µ″* + *ε″µ′*)^2^]^1/2^}^1/2^) of the samples are further compared in Figure 8d and Figure S23 (Supporting Information).^[^ ^54^ ^]^ All the *α* values exhibit an increasing trend as the frequency increases. Among the three samples, CoAl/Fe_0.8_Co_0.2_S has the largest *α* values in the low and medium frequency range, implying the strongest EMW absorption ability in these frequency ranges. Impedance matching (*Z* = (*µ_r_*/*ε_r_*)^1/2^tanh|*j*2*πfd*(*µ_r_ε_r_*)^1/2^/*c*|) determines whether EM waves can enter the material as much as possible.^[^ ^55^ ^]^ As shown in Figure 8e, the *Z* values of the three samples are close to 1, indicating that most EMW can enter samples, which benefits the attenuation of EMW inside the materials. The comparison of impedance matching for CoAl/Fe_0.8_Co_0.2_S at different matching thicknesses is revealed in Figure 8f. Apparently, the *Z* values of CoAl/Fe_0.8_Co_0.2_S in the low and middle‐frequency ranges are closer to 1, which leads to stronger EMW absorption in these ranges. The *Z* values of CoAl/Fe_0.8_Co_0.2_S with different vulcanization times are displayed in Figure S24 (Supporting Information), which shows that CoAl/Fe_0.8_Co_0.2_S with vulcanization times of 30 and 60 min has better impedance matching.

To the best of our knowledge, the EMW absorption ability of the constructed solid‐solution‐type sulfides is currently the best performance in the frequency range of 2–6 GHz, as compared in Figure S25 and Table S1 (Supporting Information). Especially, the *R*_L_/*d* value of CoAl/Fe_0.8_Co_0.2_S, which is commonly used to evaluate the absorption ability of thin absorbers, is much higher than those of other metal sulfide‐based absorbers (Figure 8g). In addition, it is interesting to find that CoAl/Fe_0.8_Co_0.2_S also exhibits attractive frequency selection behavior in the low‐frequency range (2–6 GHz, 5G mobile phones). When the material thickness changes from 3.95, 5.0, 6.5 to 8.45 mm, the sharp absorption peak can effectively absorb undesirable EM waves of a specific frequency (Figure 8h). This indicates the potential of solid‐solution‐type sulfides in smart switches for functional EMW absorption in 5G mobile phones.

**Figure 9:**
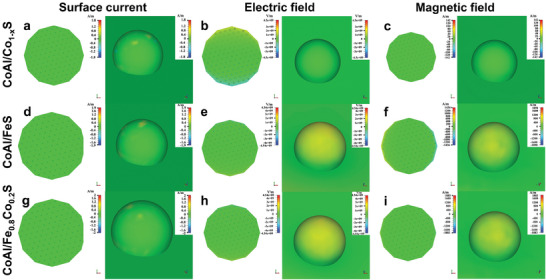
a,d,g) Surface current, b,e,h) electric‐field, and c,f,i) magnetic‐field distributions of a–c) CoAl/Co_1‐x_S, d–f) CoAl/FeS, and g–i) CoAl/Fe_0.8_Co_0.2_S at a frequency of 2 GHz.

To identify the advantages of solid‐solution‐type sulfides in EM response compared to monometallic sulfides, computer simulation technology (CST) program was used to simulate the surface current, electric field, and magnetic field distributions of CoAl/Co_1‐x_S, CoAl/FeS, and CoAl/Fe_0.8_Co_0.2_S at different frequencies (2, 10, 18 GHz),^[^ ^56^, ^57^ ^]^ as described in the Supporting Information. Briefly, CoAl nanoplates loaded with Co_1‐x_S, FeS, and Fe_0.8_Co_0.2_S nanoparticles serve as three structural configurations. Compared with CoAl/Co_1‐x_S and CoAl/FeS, CoAl/Fe_0.8_Co_0.2_S shows higher intensity in electric field and magnetic field (**Figure** **9**), which is consistent with the stronger EM response of solid‐solution‐type sulfides. Figures S26 and S27 (Supporting Information) indicate that the intensity of the electric field and magnetic field is weaker at higher frequencies (10 and 18 GHz). This result reveals that the sample enables stronger magnetic dissipation ability at higher frequencies, which is consistent with the above permeability results. Furthermore, the electric field and magnetic field strengths of metal sulfide nanoparticles are significantly higher than those of CoAl nanoplates, confirming the main contribution of metal sulfides. In addition, CoAl/Fe_0.8_Co_0.2_S exhibits a relatively smaller surface current density, indicating its appropriate matching performance.^[^ ^28^ ^]^ Apparently, nanoplates have better matching performance than nanoparticles, which is due to the low conductivity of CoAl. The surface current of the sample is stronger along the radial direction of the nanoparticles, which can be attributed to the radial polarized vortex EMW generated under “off‐resonance” conditions.^[^ ^28^, ^58^, ^59^ ^]^ Notably, CoAl/Fe_0.8_Co_0.2_S inherits a stronger electric field at the edge of the nanoplate (Figure S28, Supporting Information), which is due to the good interface polarization at the edge. At the same time, the samples exhibit a stronger magnetic field at the edge of the nanoplate (Figures S29–S31, Supporting Information), which can be attributed to the asymmetric magnetic distribution at the edge of the nanoplate. These simulation results demonstrate the better dielectric and matching properties of solid‐solution‐type sulfides, as well as the excellent EM characteristics at the edges and interfaces of the CoAl/Fe_0.8_Co_0.2_S heterostructure.

**Figure 10:**
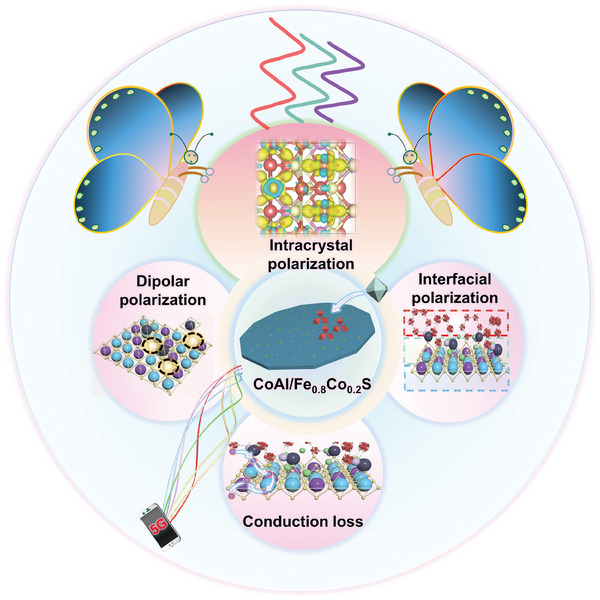
EMW absorption mechanism for CoAl/Fe_0.8_Co_0.2_S heterostructure.

Based on the above systematic investigation, the EMW absorption mechanism of CoAl/Fe_0.8_Co_0.2_S heterostructure has been proposed, as shown in **Figure** **10**. The exsolution and recombination processes led to the formation of solid‐solution‐type sulfide nanoparticles, thereby enriching the component and structure of the absorber. This enrichment, coupled with the doping of rich N heteroatoms generated during the sulphurization process, and the formation of numerous defects after the metal ions dissolution, make the material exhibit great dipolar polarization.^[^ ^60^ ^]^ This potential is essential for enhancing EMW absorption performance. Importantly, the strong intracrystal polarization in solid‐solution‐type sulfides is beneficial for improving dielectric properties in high‐frequency EM fields. Moreover, the dense arrangement of solid‐solution‐type sulfide (Fe_0.8_Co_0.2_S) nanoparticles on the CoAl‐related 2D nanosheet matrix material enhances its conduction network, thereby improving conduction loss characteristics.^[^ ^61^ ^]^ The high electronic conductivity of solid‐solution‐type sulfide can also optimize conduction loss. At the same time, the rich composite structure significantly increases the electron transmission path, leading to enhanced conduction loss. Therefore, intracrystal polarization loss and conduction loss play important roles in improving the EMW absorption of solid‐solution‐type sulfide. In addition, multiple heterogeneous interfaces on CoAl/Fe_0.8_Co_0.2_S amplify its interfacial polarization potential,^[^ ^62^, ^63^ ^]^ also enhancing EMW absorption performance. The exsolution and recombination of metal sulfide nanoparticles in CoAl/Fe_0.8_Co_0.2_S heterostructure achieve abundant polarization relaxation, including natural resonance, eddy current loss, and exchange resonance. Overall, the rich component and structure, strong intracrystal polarization, and optimized impedance matching contribute to its excellent EMW absorption capacity.

## 3 Conclusion

In summary, a precise sulphurization strategy was executed for the in situ exsolution of metal ions from in 2D CoAl‐LDH nanosheets and spindle‐like MIL‐88A nanoparticles. By a competitive reaction with S^2−^, the recombination route was carried out to form solid‐solution‐type sulfides (Fe_0.8_Co_0.2_S) on the CoAl matrix, in order to develop a CoAl/Fe_0.8_Co_0.2_S heterostructure that inherits rich components and structures. The rich interfaces and polarization centers formed in heterostructures greatly contribute to their polarization relaxation and amplify their dielectric loss capability. Importantly, the intracrystal polarization in solid‐solution‐type sulfides has been proposed and demonstrated, which greatly contributes to improving the dielectric loss ability. Furthermore, magnetic components in heterostructures demonstrate a multifaceted magnetic loss mechanism, including natural resonance, eddy current loss, and exchange resonance. This intricate magnetic behavior synergizes with the dielectric properties to fine‐tune the impedance matching of heterostructures. Accordingly, the developed CoAl/Fe_0.8_Co_0.2_S heterostructure enables the highest EMW absorption performance within the low‐frequency band (2–6 GHz), with an *R*_L_ value of ─65.26 dB at a matching thickness of 3.5 mm. CoAl/Fe_0.8_Co_0.2_S heterostructure also exhibits frequency selection behavior in the practical frequency range used by 5G mobile phones. In a broader context, this work paves a viable route for employing solid‐solution‐type sulfides in the realm of EMW absorbers. Additionally, by harnessing the potential of exsolution and recombination for metal ions, this work opens up the possibility of designing and optimizing absorbers with enhanced EMW absorption performance.

## 4 Experimental Section

### Chemicals

Cobalt nitrate hexahydrate (Co(NO_3_)_2_·6H_2_O, purity 98%), aluminum chloride hexahydrate (AlCl_3_·6H_2_O, purity 98%), hexahydrate iron chloride (FeCl_3_·6H_2_O, purity 99%), N,N‐dimethylformamide (C_3_H_7_NO, DMF, purity 99.5%), fumaric acid (C_4_H_4_O_4_, FA, purity 98%), urea (CH_4_N_2_O, purity 99%), and thiourea (CH_4_N_2_S, purity 99%) were procured from Sinopharm Chemical Reagent Co., Ltd. These chemicals were used directly in their purchased state without the need for additional purification steps.

### Synthesis of CoAl‐LDH

The 1.1641 g of Co(NO_3_)_2_·6H_2_O, 0.4829 g of AlCl_3_·6H_2_O, and 0.8408 g of urea were meticulously dissolved in 400 mL of deionized water. The amalgamation was subjected to stirring at room temperature for a duration of 15 min, following which a refluxing process was initiated within an oil bath set at 97 °C. This refluxing stage persisted for 48 h. Throughout this refluxing interval, urea exhibited gradual dissolution, thereby engendering a mildly alkaline milieu. This alkaline environment facilitated the proliferation of CoAl‐LDH. After the refluxing process, a regimen of free cooling ensued, accompanied by repeated washing using deionized water. Subsequently, the obtained product, characterized by a pink hue, was subjected to room‐temperature drying. This multifaceted procedure culminated in the formation of pink‐colored CoAl‐LDH.

### Synthesis of CoAl‐LDH/MIL‐88A

The 0.08 g of CoAl‐LDH and 1.0812 g of FeCl_3_·6H_2_O was dispersed in 10 mL of DMF. The mixture underwent stirring at room temperature for 10 min, followed by a 5 min sonication process. Subsequently, 0.4643 g of FA was added to another 10 mL of DMF, which was then subjected to stirring at room temperature for 15 min. The resultant FA‐infused DMF solution was amalgamated with the initial CoAl‐LDH and FeCl_3_·6H_2_O mixture. The combined solution was subjected to an additional 10 min stirring phase before introducing into the high‐pressure reactor. Within the high‐pressure reactor, the reaction was conducted at 100 °C for 4 h. Upon completion of the reaction, the solution was allowed to cool and was subsequently subjected to multiple washes using DMF and deionized water. Subsequently, the powder was placed within a constant‐temperature drying oven and subjected to vacuum drying at 40 °C for 12 h. This comprehensive procedure ultimately achieved brown‐colored CoAl‐LDH/MIL‐88A.

### Synthesis of CoAl/Fe_0.8_Co_0.2_S

The conversion of CoAl‐LDH/MIL‐88A into CoAl/Fe_0.8_Co_0.2_S was achieved through a vulcanization process. Specifically, a quantity of 0.5 g of thiourea was positioned at the front end of a tubular furnace, while CoAl‐LDH/MIL‐88A was placed at the rear end. The transformation to CoAl/Fe_0.8_Co_0.2_S was effectuated by subjecting the arrangement to pyrolysis at a temperature of 700 °C, within an N_2_ atmosphere flowing at a rate of 200 mL min^−1^. This pyrolysis phase extended for 30 min, with a heating rate of 5 °C min^−1^. For comparative analysis, two additional sulphurization durations were executed, spanning 10 and 60 min, respectively. In addition, CoAl‐LDH and MIL‐88A were also vulcanized to obtain CoAl/Co_1‐x_S and NC/FeS, respectively.

### Materials Characterization

The investigation into the crystal structure of samples was conducted through the utilization of an X‐ray diffraction (XRD) spectrometer (D8 Advance, Bruker Germany), employing Cu Kα radiation (λ = 1.5418 Å, 40 kV, 30 mA). In‐depth analysis of the surface element valence states was performed using X‐ray photoelectron spectroscopy (XPS) through the employment of a Thermo Escalab 250Xi instrument. For comprehensive insights into the characteristics of the material, both the surface morphology and internal composition were scrutinized. Field emission scanning electron microscopy (FESEM) was carried out using an SU‐8010 microscope from Hitachi, Japan. In conjunction with the FESEM device, energy dispersive X‐ray spectroscopy (EDS, Oxford, Xplore) was employed to detect the elemental distribution across the sample Furthermore, the transmission electron microscopy (TEM) technique was harnessed, facilitated by the FEI Talos F200x G2 instrument, which additionally featured Bruker super‐X EDS capabilities. The hysteresis loop of the sample can be obtained using a vibrating sample magnetometer (VSM, Lake Shore 7404).

### Electromagnetic Parameters Test

Based on the coaxial line theory, the electromagnetic parameters of the samples were measured using the vector network analyzer (Agilent E5071C). The absorption ring to be tested is made of sample and paraffin in a mass ratio of 6:4, with an inner diameter of 3.04 mm and an outer diameter of 7.0 mm. The coaxial line method is used to measure the electromagnetic parameters of the material (real part of dielectric constant (*ε′*) and magnetic permeability (*µ*′), the imaginary part of dielectric constant (*ε′′*) and permeability (*µ′′*)), with a frequency range of 2–18 GHz. Based on the transmission line theory, the reflection loss (*R*_L_) values were calculated for different thicknesses and corresponding frequencies.

(3) $$Z_{\mathrm{in}}=Z_{0}{\left(\mu_{r}/\varepsilon_{r}\right)}^{1/2}\tanh\left[j\left(2\pi ft/c\right){\left(\mu_{r}\varepsilon_{r}\right)}^{1/2}\right]$$

(4) $$R_{L}=\;\;20\mathrm{lo}g_{10}\left|\left(Z_{\mathrm{in}}-Z_{0}\right)/\left(Z_{\mathrm{in}}+Z_{0}\right)\right|$$

where *c* is the speed of light, *f* is the frequency, *t* is the matching thickness, *Z*_in_ is the input impedance, *Z*_0_ is the spatial free impedance, *ε*_r_ is the complex permittivity, and *µ*_r_ is the complex permeability.

### Calculation Process

To further investigate the EMW absorption mechanism of CoAl/Fe_0.8_Co_0.2_S heterostructures, computer simulation technology (CST) was used to simulate the frequency‐dependent electric‐field, magnetic‐field, and surface current distributions of CoAl/Fe_0.8_Co_0.2_S heterostructure (see details in the Supporting Information).

To further reveal the charge density and the density of states (DOS) of the absorbers, density functional theory (DFT) calculations were conducted (see details in the Supporting Information).

## Conflict of Interest

The authors declare no conflict of interest.

## Supporting information

Supporting Information

## Data Availability

The data that support the findings of this study are available from the corresponding author upon reasonable request.
