# Supplementary material for: In Situ Exsolution‐Prepared Solid‐Solution‐Type Sulfides with Intracrystal Polarization for Efficient and Selective Absorption of Low‐Frequency Electromagnetic Wave
Source: Adv Sci (Weinh). 2024 Jul 16;11(35):2403723. doi: 10.1002/advs.202403723 (PMC11425237; doi:10.1002/advs.202403723)
Supplement: Supplementary file 1 — Supporting Information [file ADVS-11-2403723-s001.docx]

Supporting Information

In-situ exsolution-prepared solid-solution-type sulfides with intracrystal polarization for efficient and selective absorption of low-frequency electromagnetic wave

Xiaojun Zeng,* Tianli Nie, Chao Zhao, Yangfeng Gao,* and Xiaofang Liu*

**Synthesis of CoFe-LDH and CoFe-LDH derived sulfides**

Based on the previsou work, CoFe-LDH was prepared.^[1]^ Co(NO_3_)_2_·6H_2_O (2.9103 g) and Fe(NO_3_)_3_·9H_2_O (2.02 g) were added into 100 mL of deionized water. After stirring for 30 min, 3.6046 g of urea was dissolved in the above solution under magnetic stirring at 96 ℃ for 48 h. The resulting CoFe-LDH was centifuged, washed with distilled water, and dried at 45 ℃ for 24 h. The vulcanization process of CoFe-LDH is same as that of CoAl-LDH, and the resulting products was labeled as CoFe-LDH derived sulfides. Specifically, the transformation to sulfides was achieved by pyrolysis at 700 ℃ for 30 min. The N_2_ flow rate was 200 mL min^-1^, and the heating rate was 5 ℃ min^-1^.

**RCS simulation process**

CST Studio Suite 2022 was employed to simulate the Radar Cross-Section (RCS) of CoAl/Fe_0.8_Co_0.2_S heterostructure. Typically, the perfect electric conductor (PEC) model with a base area of 200 mm × 200 mm and a thickness of 3.52 mm is established, while the PEC surface (200 mm × 200 mm) is coated with a wave-absorbing material with a thickness set to 3.52 mm. The model plate was placed on the XOY plane, the linearly polarized plane electromagnetic wave was incident from the positive direction of the Z axis to the negative direction of the Z axis, and the electric polarization direction propagates along the X axis. Open boundary conditions are set in all directions, and the field monitor frequency is 6 GHz. The scattering direction is determined by theta and phi in spherical coordinates. RCS can be defined as:^[2-4]^

*σ* (dB m^2^) = 10log{(4*πS*/*λ*^2^)(|*E*_s_/*E*_i_|)}^2^

where *S*, *λ*, *E*_s_, and *E*_i_ represent the area of the simulation model, the wavelength of electromagnetic wave, the electric field intensity of scattered wave and the incident wave, respectively.

**Calculation process**

The frequency-dependent electric-field, magnetic-field, and surface current distributions are simulated by a computer simulation technology (CST) program with the following conditions.

First, set the background material to normal. The Z direction is open space. Periodic boundary conditions are applied in the X and Y directions, while absorbing boundary conditions are applied in the Z direction. The plane wave is incident with an electric field polarized in the Y direction (TE) as the excitation source. CoAl/Co_1-x_S, CoAl/FeS, and CoAl/Fe_0.8_Co_0.2_S are acted as cell unites and periodically distributed on the surface of the Cu substrate, respectively. The corresponding electric-field, magnetic-field, and surface current distributions are simulated in response to the alternating applied electromagnetic field, respectively.


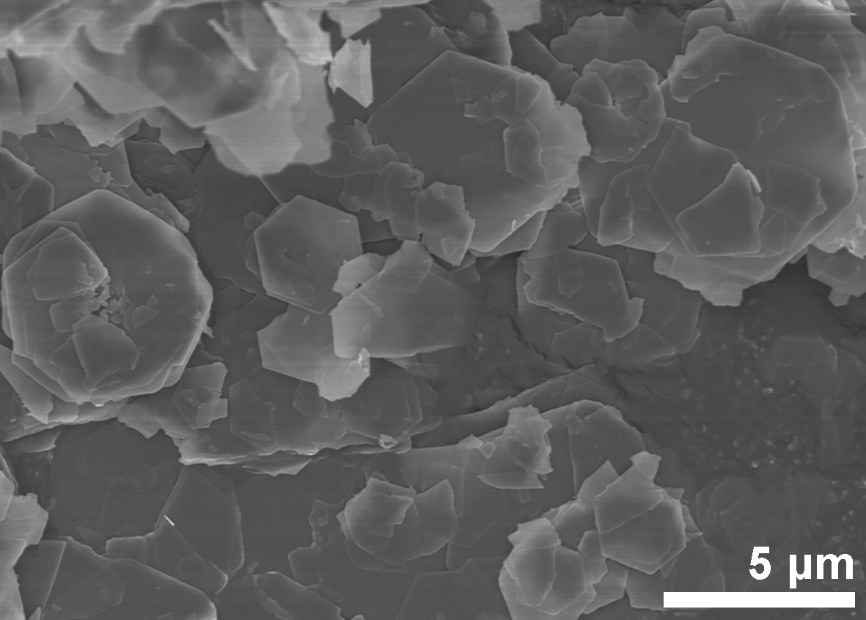


**Figure S1.** SEM images of CoAl-LDH.


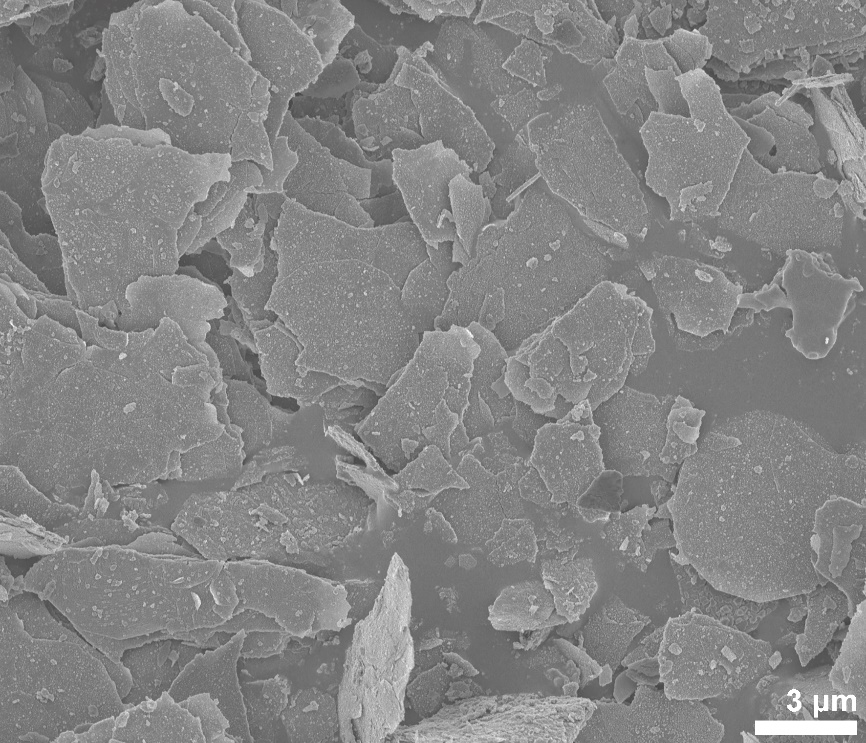


**Figure S2.** SEM images of CoAl/Co_1-x_S.


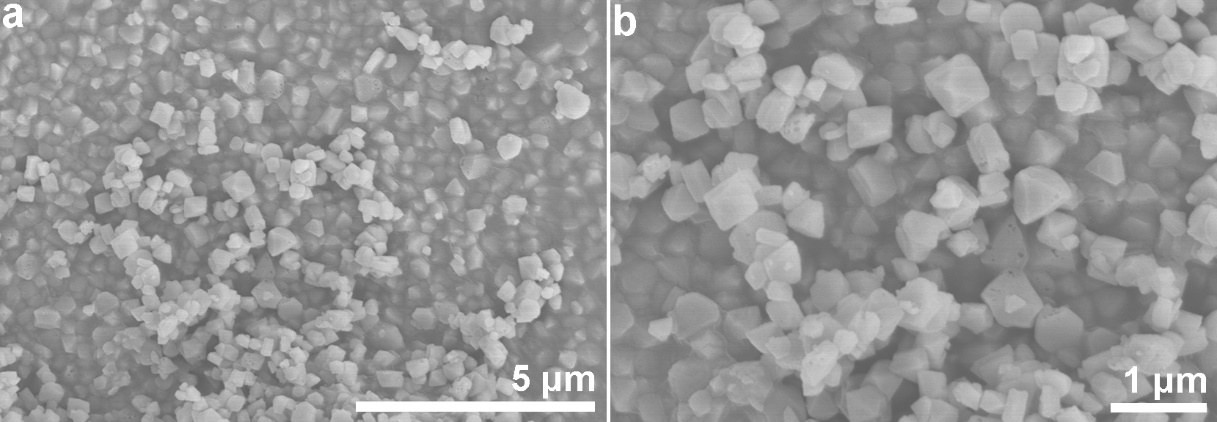


**Figure S3.** SEM images of MIL-88A.


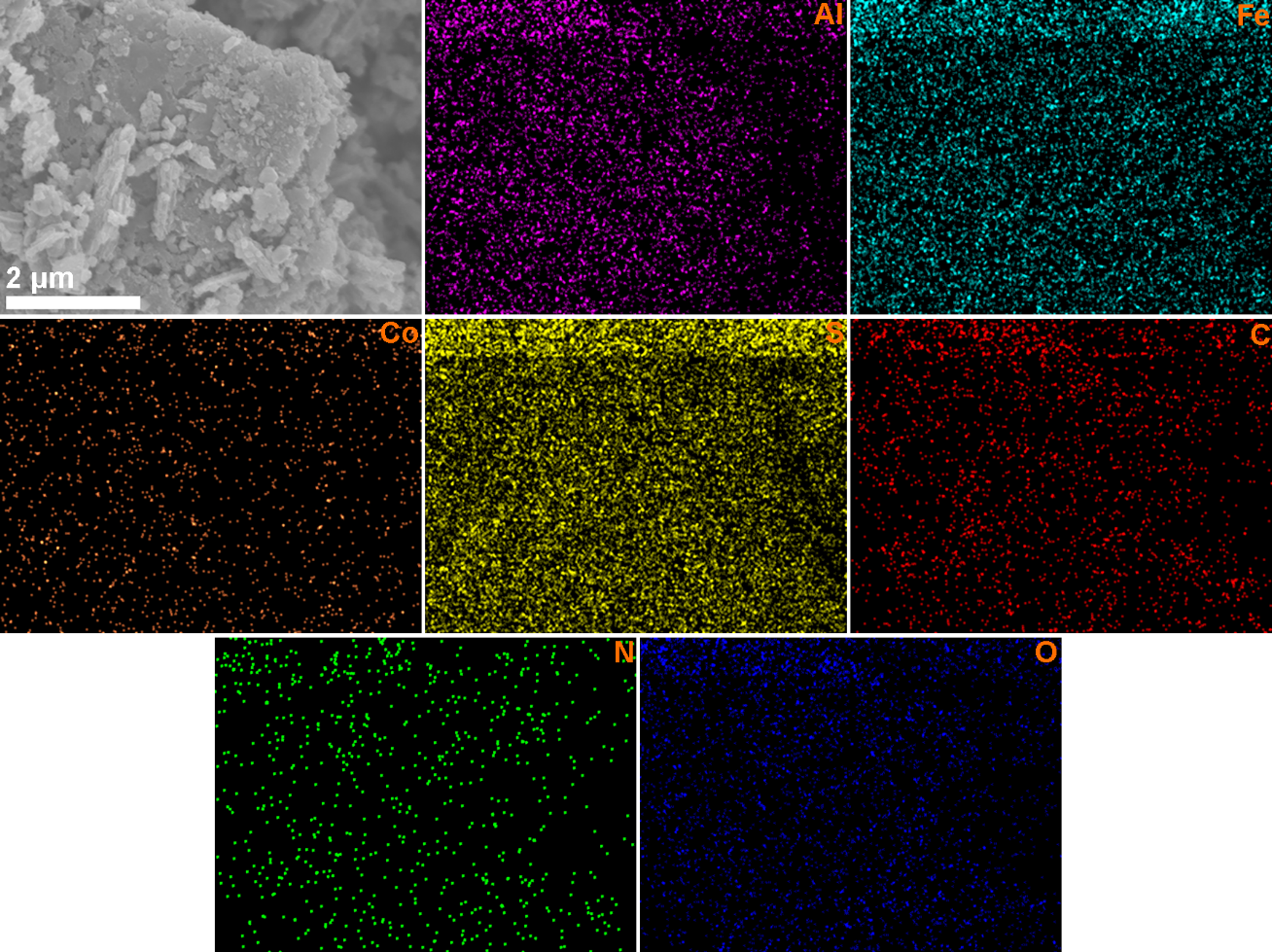


**Figure S4.** Elemental mapping image of CoAl/Fe_0.8_Co_0.2_S (obtaing from FESEM equipment).


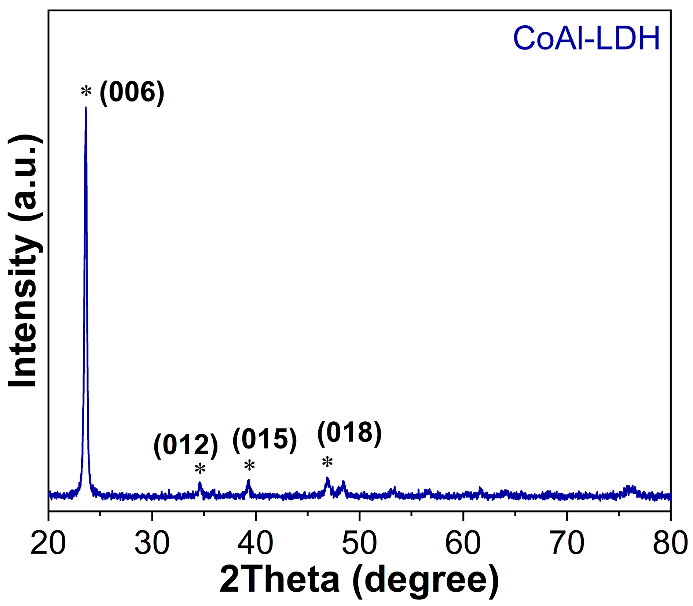


**Figure S5.** XRD patterns of CoAl-LDH.


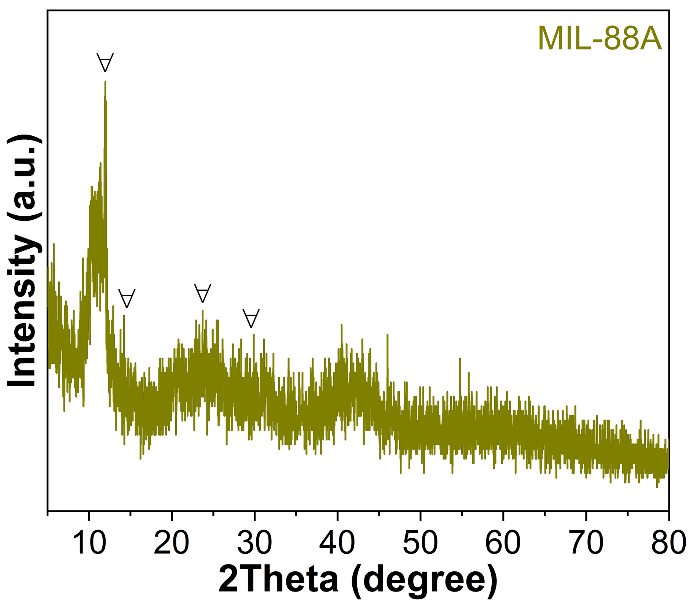


**Figure S6.** XRD patterns of MIL-88A.


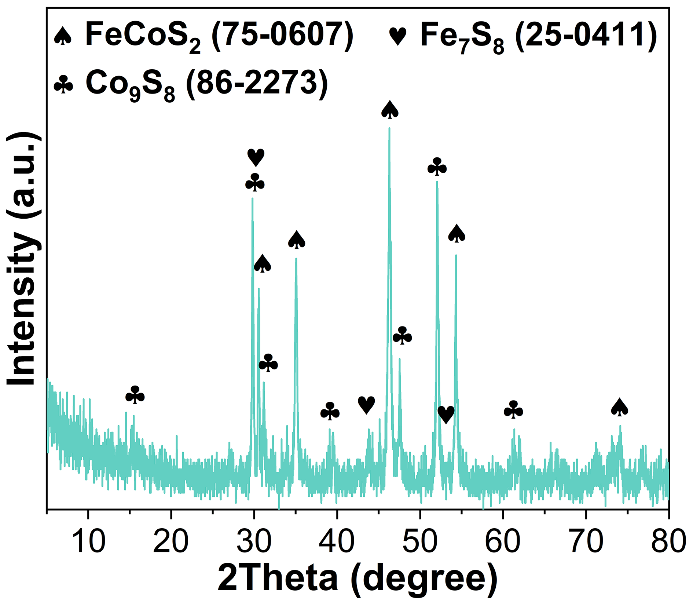


**Figure S7.** XRD patterns of CoFe-LDH derived sulfides.


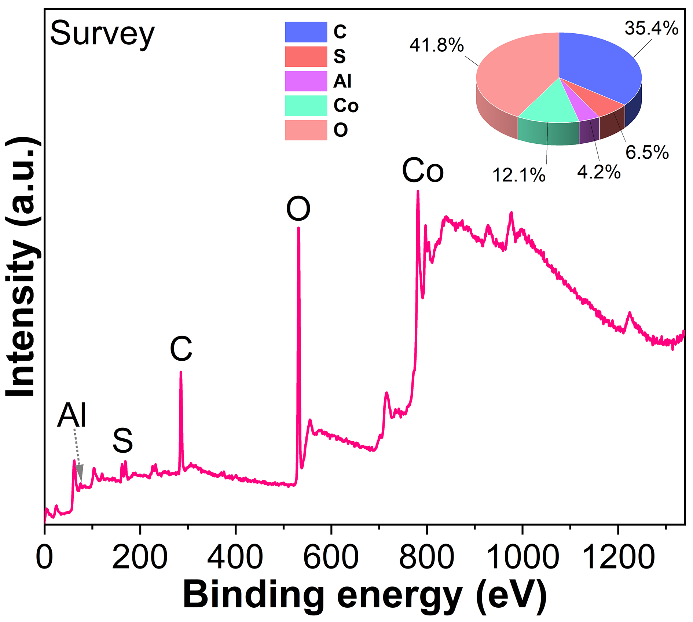


**Figure S8.** XPS survey spectra of CoAl/Co_1-x_S.


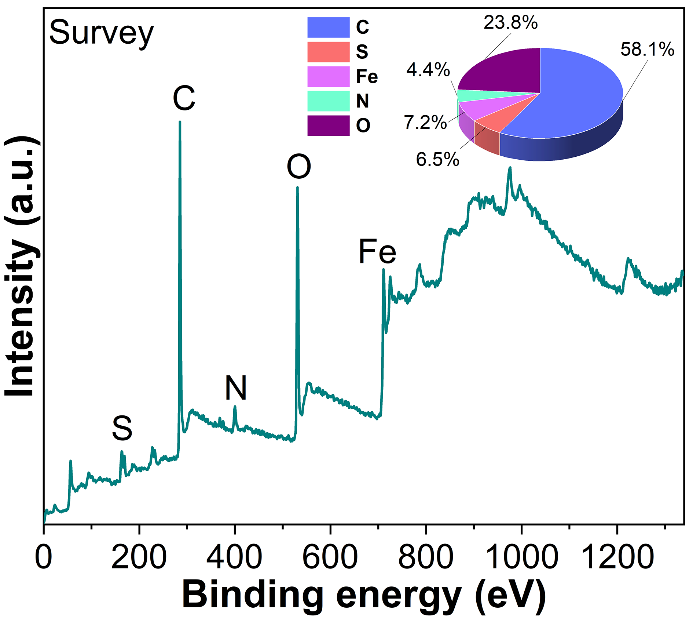


**Figure S9.** XPS survey spectra of NC/FeS.


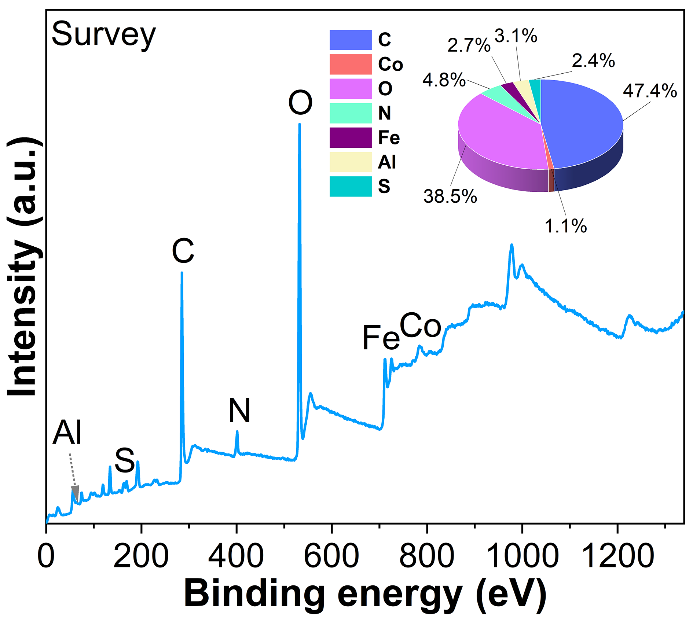


**Figure S10.** XPS survey spectra of CoAl/Fe_0.8_Co_0.2_S.


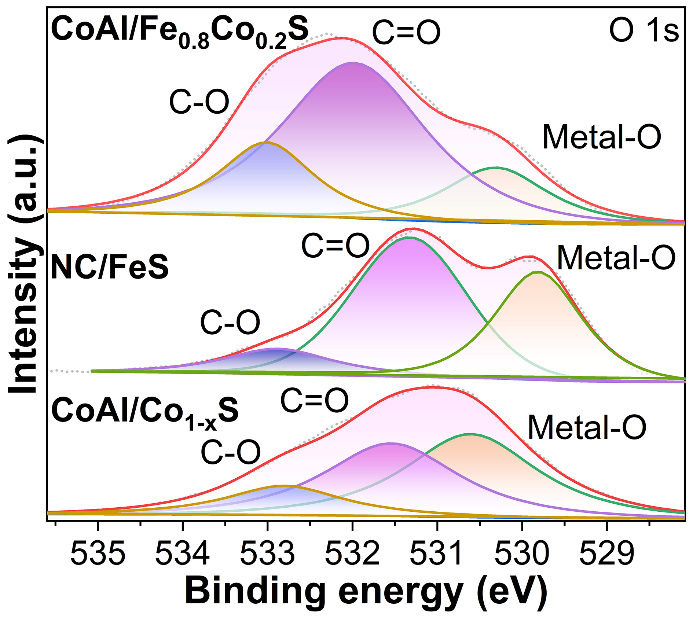


**Figure S11.** High-resolution XPS spectra of O 1s for CoAl/Co_1-x_S, NC/FeS, and CoAl/Fe_0.8_Co_0.2_S heterostructure.


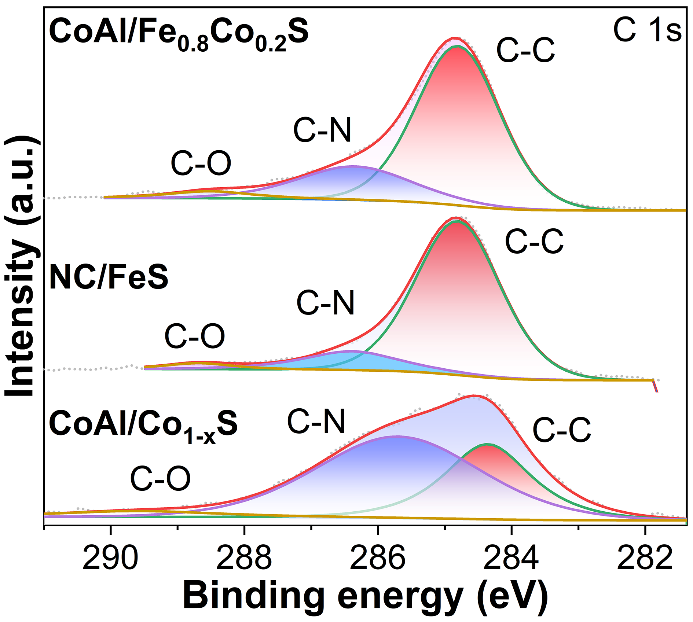


**Figure S12.** High-resolution XPS spectra of C 1s for CoAl/Co_1-x_S, NC/FeS, and CoAl/Fe_0.8_Co_0.2_S heterostructure.


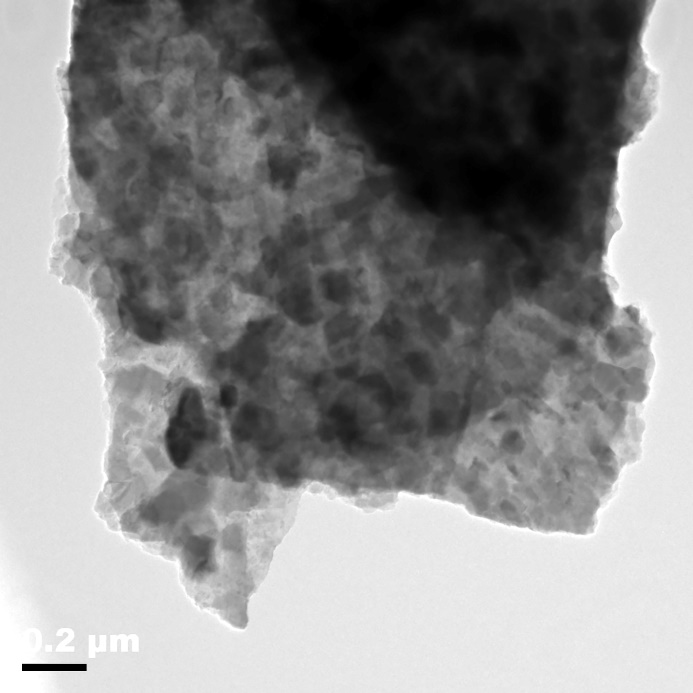


**Figure S13.** TEM images of CoAl/Co_1-x_S.


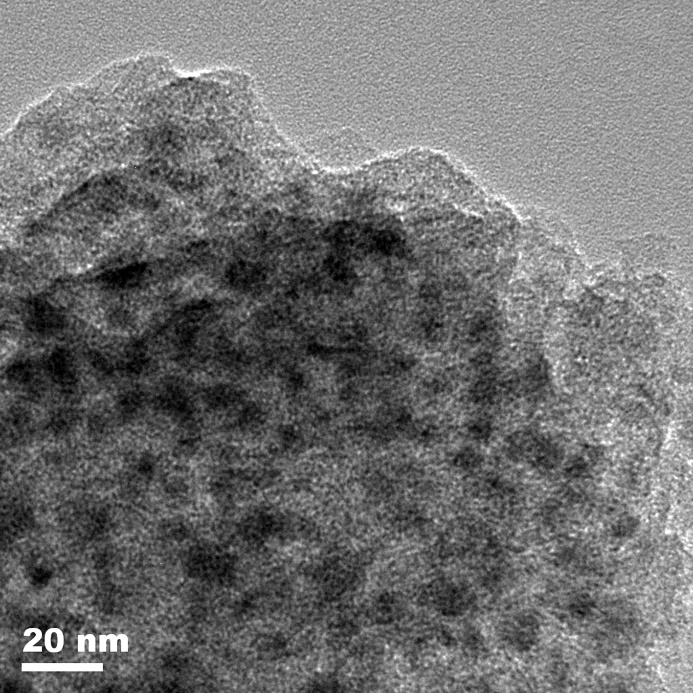


**Figure S14.** TEM images of NC/FeS.


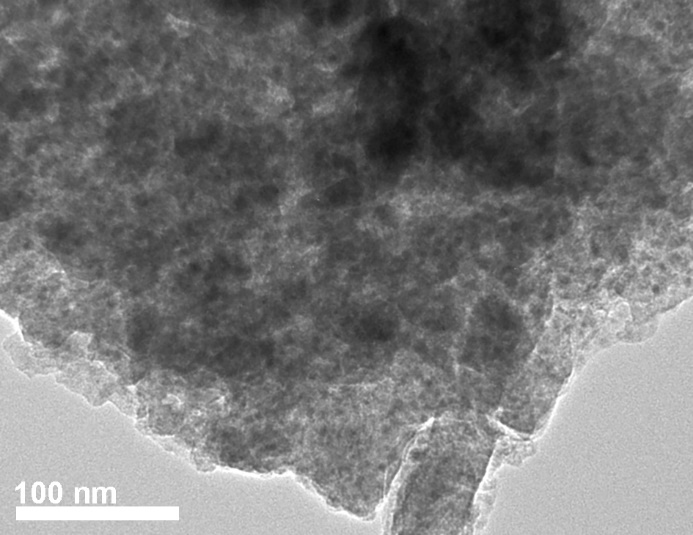


**Figure S15.** TEM images of CoAl/Fe_0.8_Co_0.2_S.


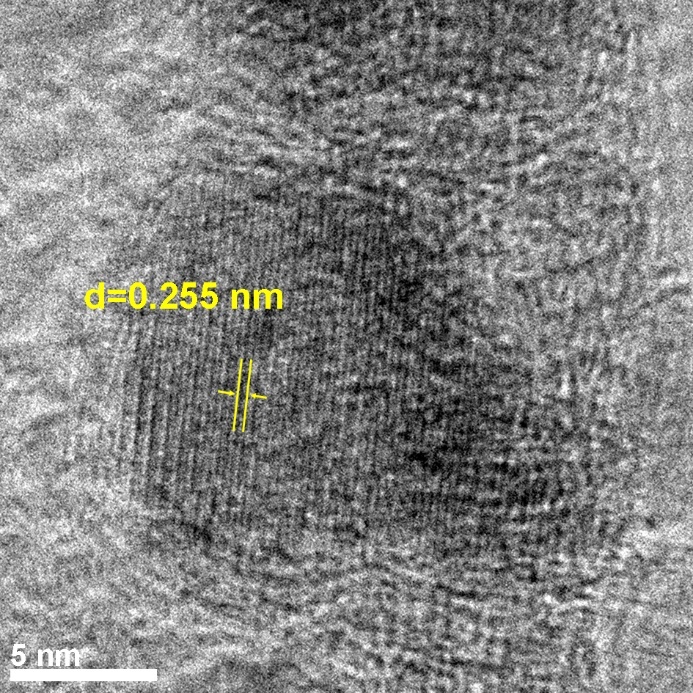


**Figure S16.** HRTEM images of CoAl/Fe_0.8_Co_0.2_S.


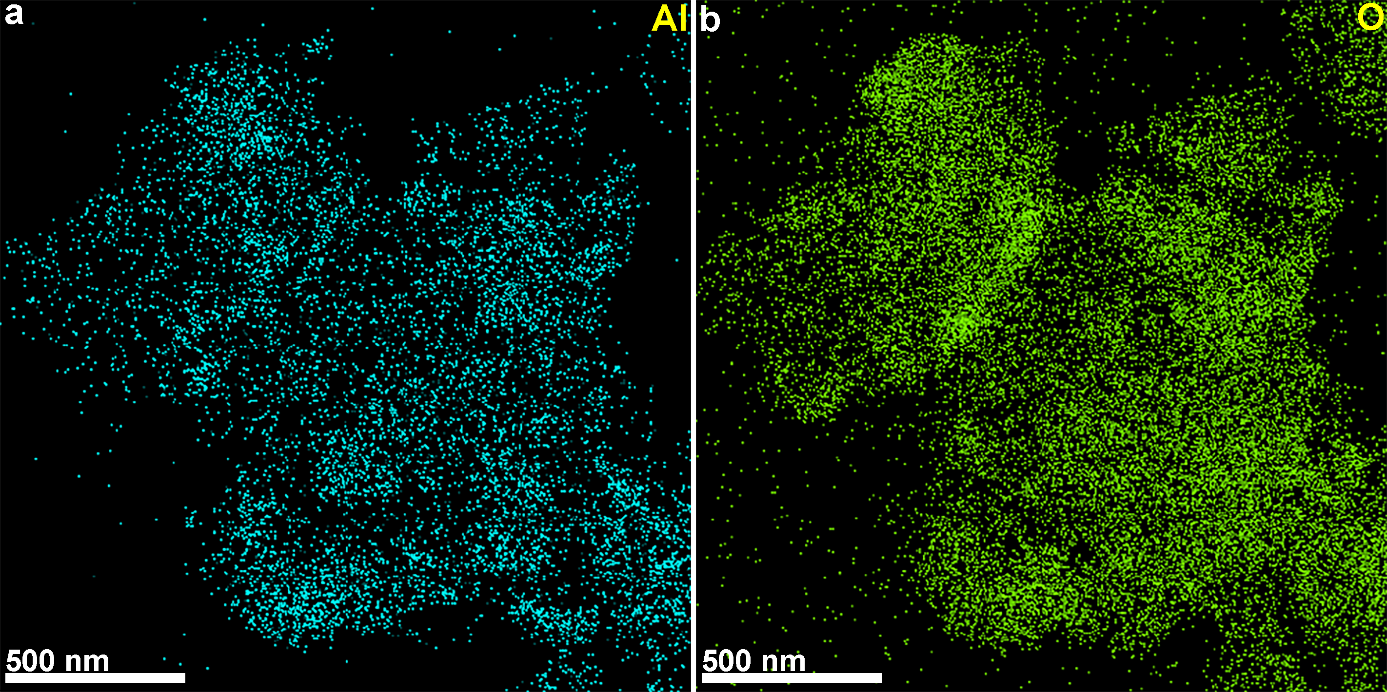


**Figure S17.** Elemental mapping image of (a) Al and (b) O elements in CoAl/Fe_0.8_Co_0.2_S (obtaing from HAADF-STEM equipment).

The influence of different vulcanization time on the EMW absorption performance of CoAl/Fe_0.8_Co_0.2_S heterostructure is displayed in Figure S18. It can be seen that too short and too long vulcanization times have a certain impact on the EMW absorption performance of materials. Even if adjusting the vulcanization time, the as-prepared solid-solution-type sulfide still maintains a certain EMW absorption capacity, as depicted in Figure S18.


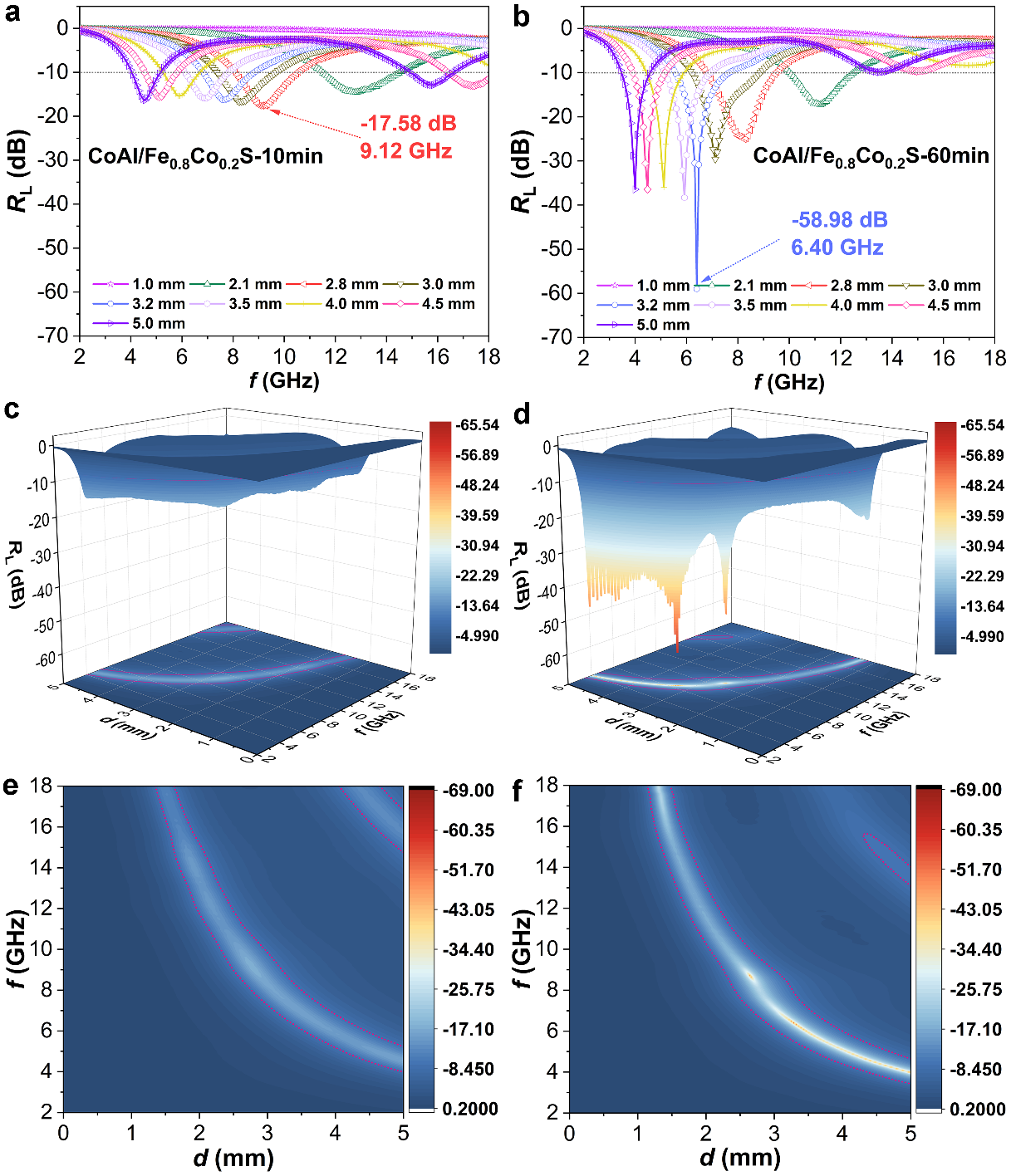


**Figure S18.** (a,b) 2D *R*_L_ curves, (c,d) 3D *R*_L_ curves, and (e,f) 2D contour maps of CoAl/Fe_0.8_Co_0.2_S heterostructure with vulcanization times of (a,c,e) 10 minutes and (b,d,f) 60 minutes.

A comparison of *ε*′ values for CoAl/Fe_0.8_Co_0.2_S with different vulcanization times, as shown in Figure S19, reveals that *ε*′ gradually increases with longer vulcanization time. A comparison of *μ*′ and *μ*″ values for CoAl/Fe_0.8_Co_0.2_S with different vulcanization times is shown in Figures S19d and 19e. The *μ*″ curves of CoAl/Fe_0.8_Co_0.2_S with longer vulcanization time (30 and 60 minutes) have resonce peaks at frequency of ~15.5 GHz, indicating the presence of exchange resonance. The formation of more solid-solution-type sulfides contributes to certain magnetic losses in the high-frequency region, balancing dielectric losses and optimizing impedance matching, thereby achieving good EMW absorption performance.


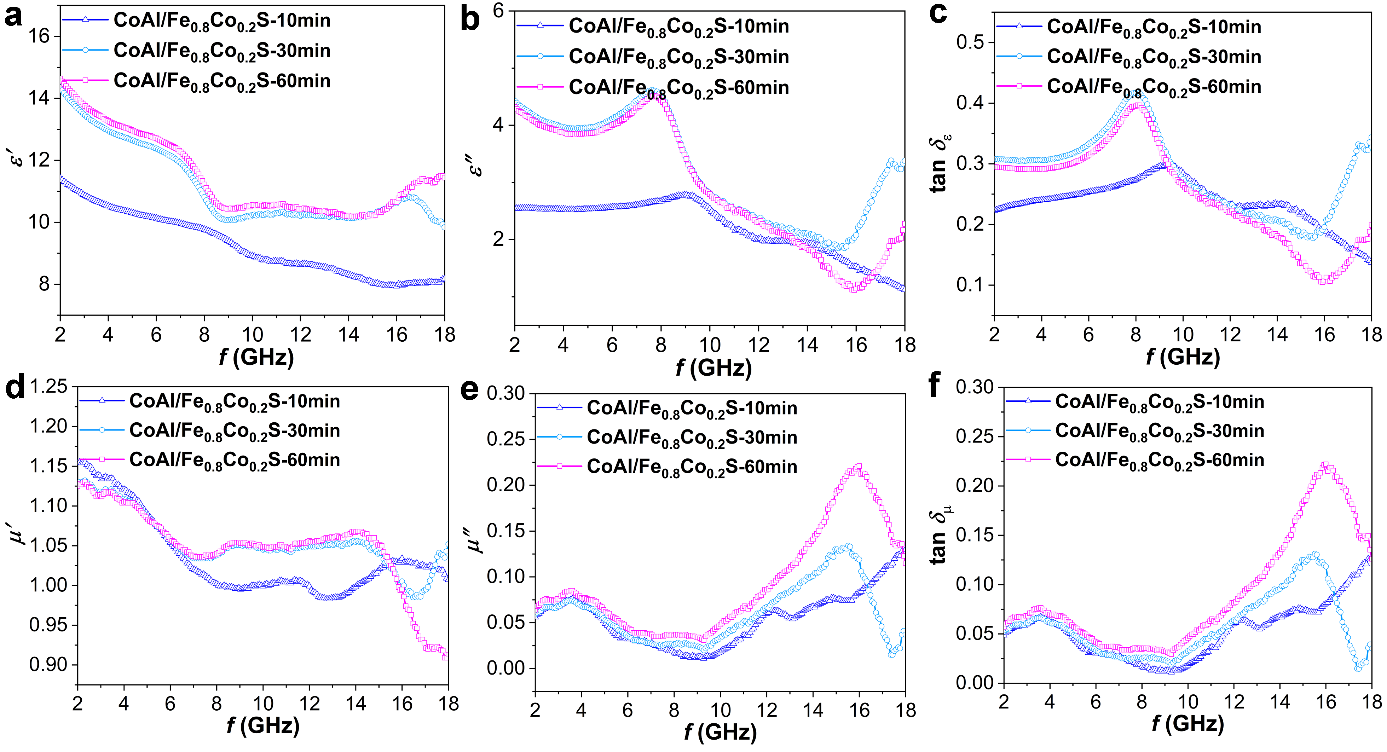


**Figure S19.** Frequency dependence of (a) *ε′*, (b) *ε′′*, (c) tan *δ*_ε_, (d) *μ′*, (e) *µ′′*, and (f) tan *δ*_µ_ curves for CoAl/Fe_0.8_Co_0.2_S heterostructure with different vulcanization times.

Figures S20c and S20d exhibit the fitted plots of *ε*′ versus *ε*″/*f* for CoAl/Fe_0.8_Co_0.2_S with different vulcanization time, and they demonstrate consistency with the results mentioned above.


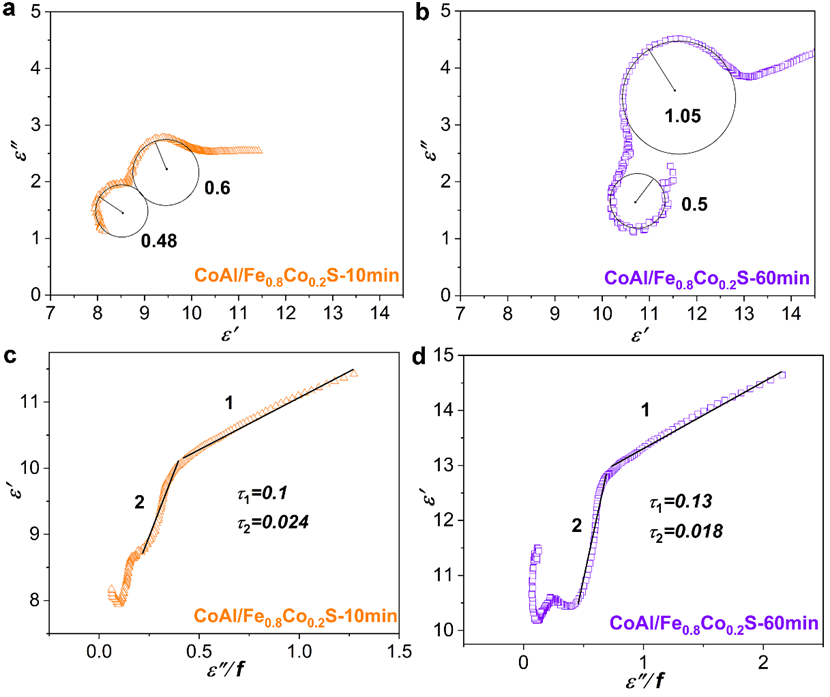


**Figure S20.** (a,b) Cole-Cole and (c,d) linear fitting curves of CoAl/Fe_0.8_Co_0.2_S heterostructure with vulcanization times of (a,c) 10 minutes and (b,d) 60 minutes.


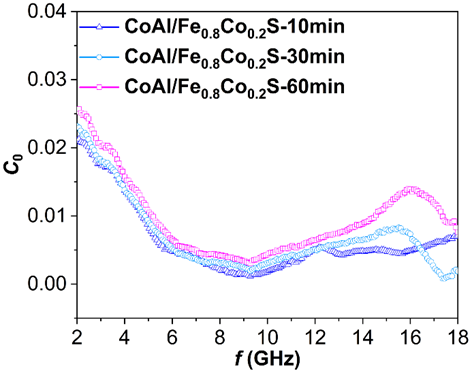


**Figure S21.** *C*_0_ values of CoAl/Fe_0.8_Co_0.2_S heterostructure with different vulcanization times.


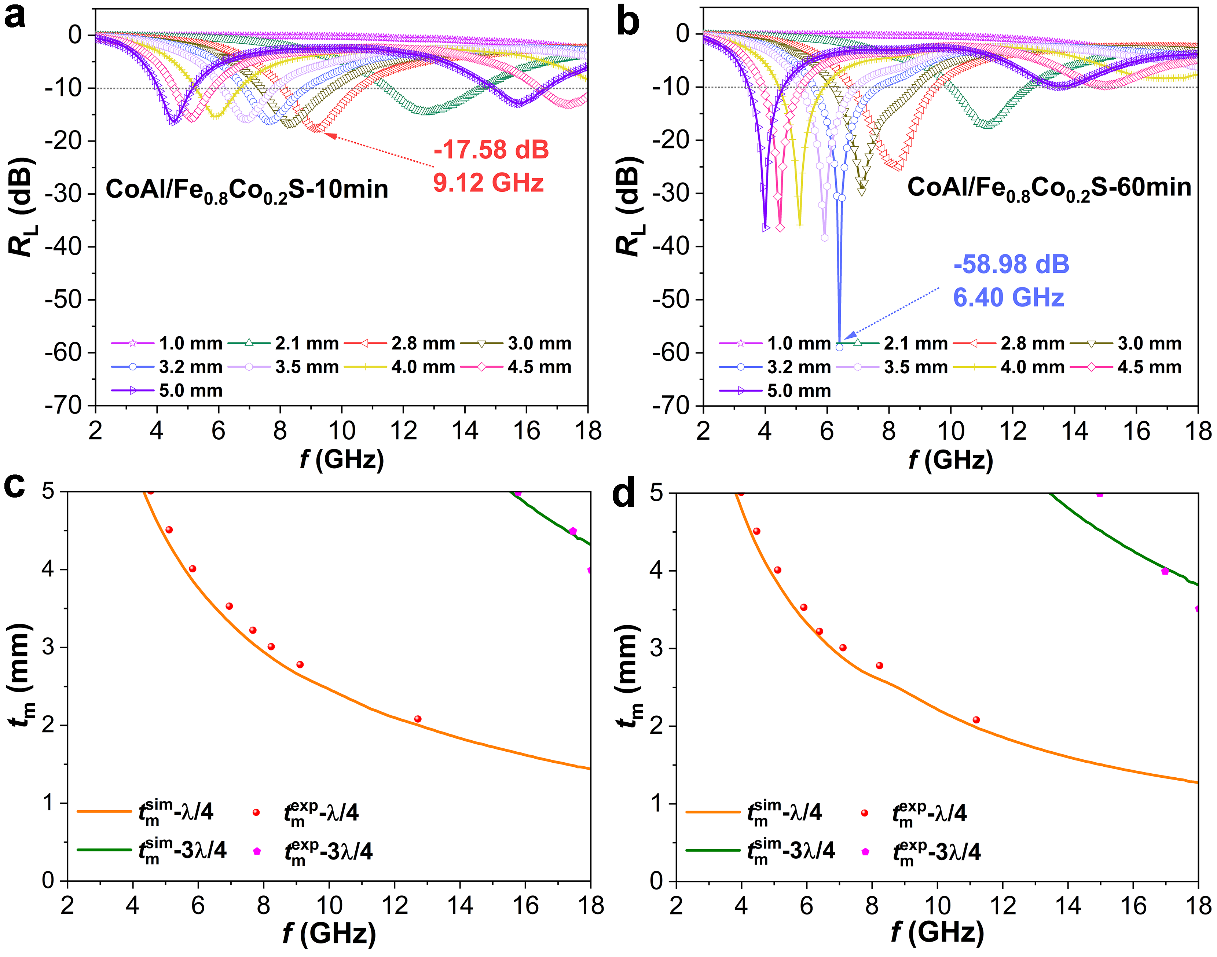


**Figure S22.** Simulation of *t*_m_ (*t*_m_^sim^) vs. *f*_m_ curves of CoAl/Fe_0.8_Co_0.2_S heterostructure with vulcanization times of (a,c) 10 minutes and (b,d) 60 minutes.

Figure S23 shows the *α* values for CoAl/Fe_0.8_Co_0.2_S with different vulcanization times. It is observed that the *α* values increase with the frequency, and a longer vulcanization time leads to higher *α* values, signifying that the vulcanization time affects the attenuation ability.


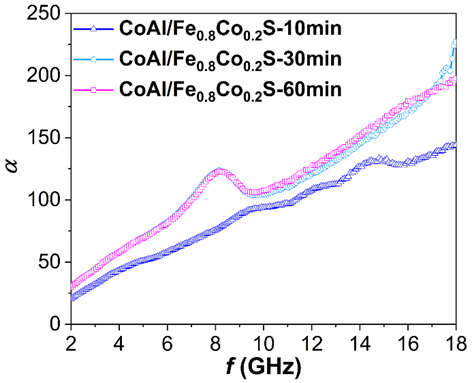


**Figure S23.** *α* values of CoAl/Fe_0.8_Co_0.2_S heterostructure with different vulcanization times.


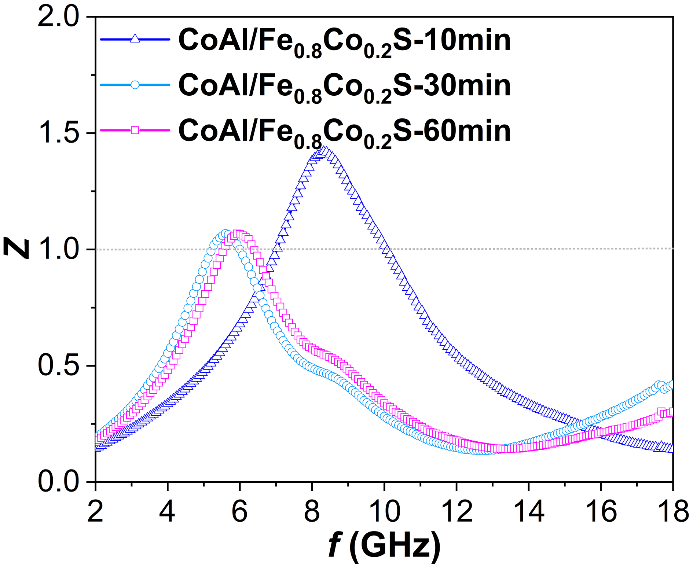


**Figure S24**. *Z* values of CoAl/Fe_0.8_Co_0.2_S heterostructure with different vulcanization times at a matching thickness of 3.5 mm.


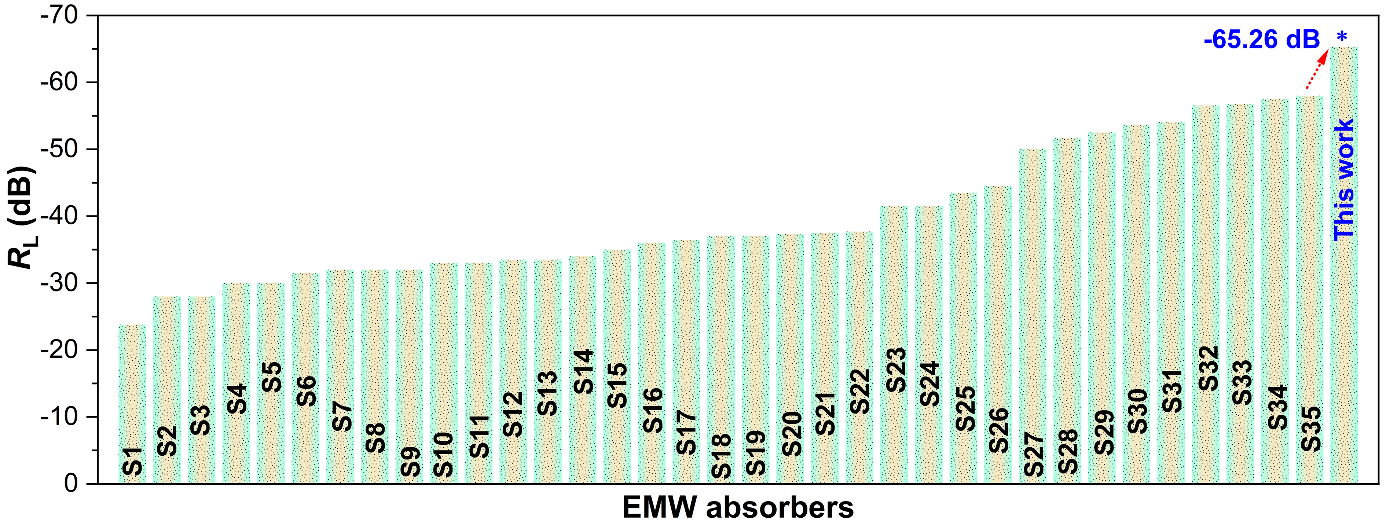


**Figure S25**. Comparison of 2D *R*_L_ values for CoAl/Fe_0.8_Co_0.2_S and advanced metal sulfide-based absorbers in the frequency range of 2-6 GHz.


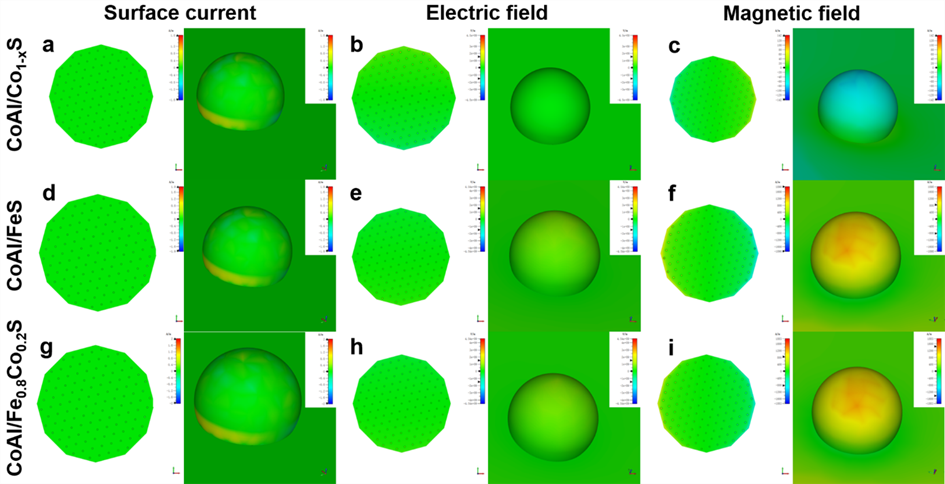


**Figure S26.** (a,d,g) Surface current, (b,e,h) electric-field, and (c,f,i) magnetic-field distributions of (a-c) CoAl/Co_1-x_S, (d-f) CoAl/FeS, and (g-i) CoAl/Fe_0.8_Co_0.2_S at frequency of 10 GHz.


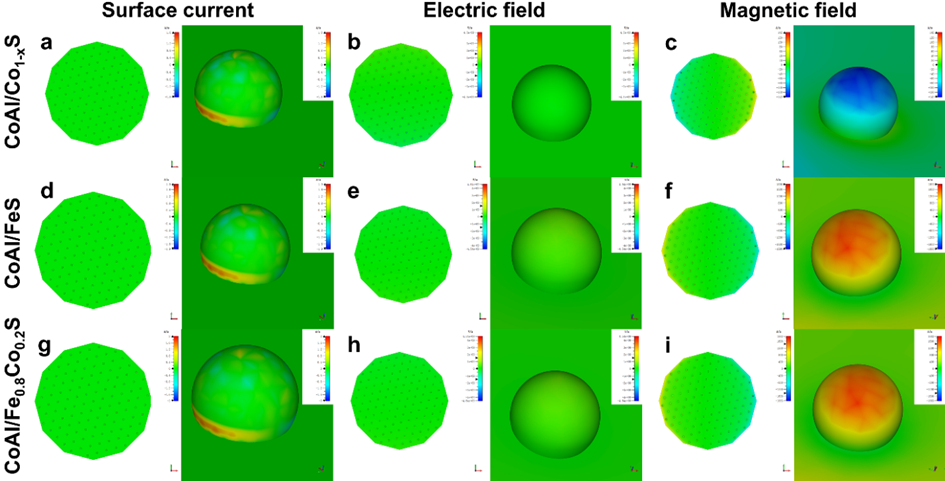


**Figure S27.** (a,d,g) Surface current, (b,e,h) electric-field, and (c,f,i) magnetic-field distributions of (a-c) CoAl/Co_1-x_S, (d-f) CoAl/FeS, and (g-i) CoAl/Fe_0.8_Co_0.2_S at frequency of 18 GHz.


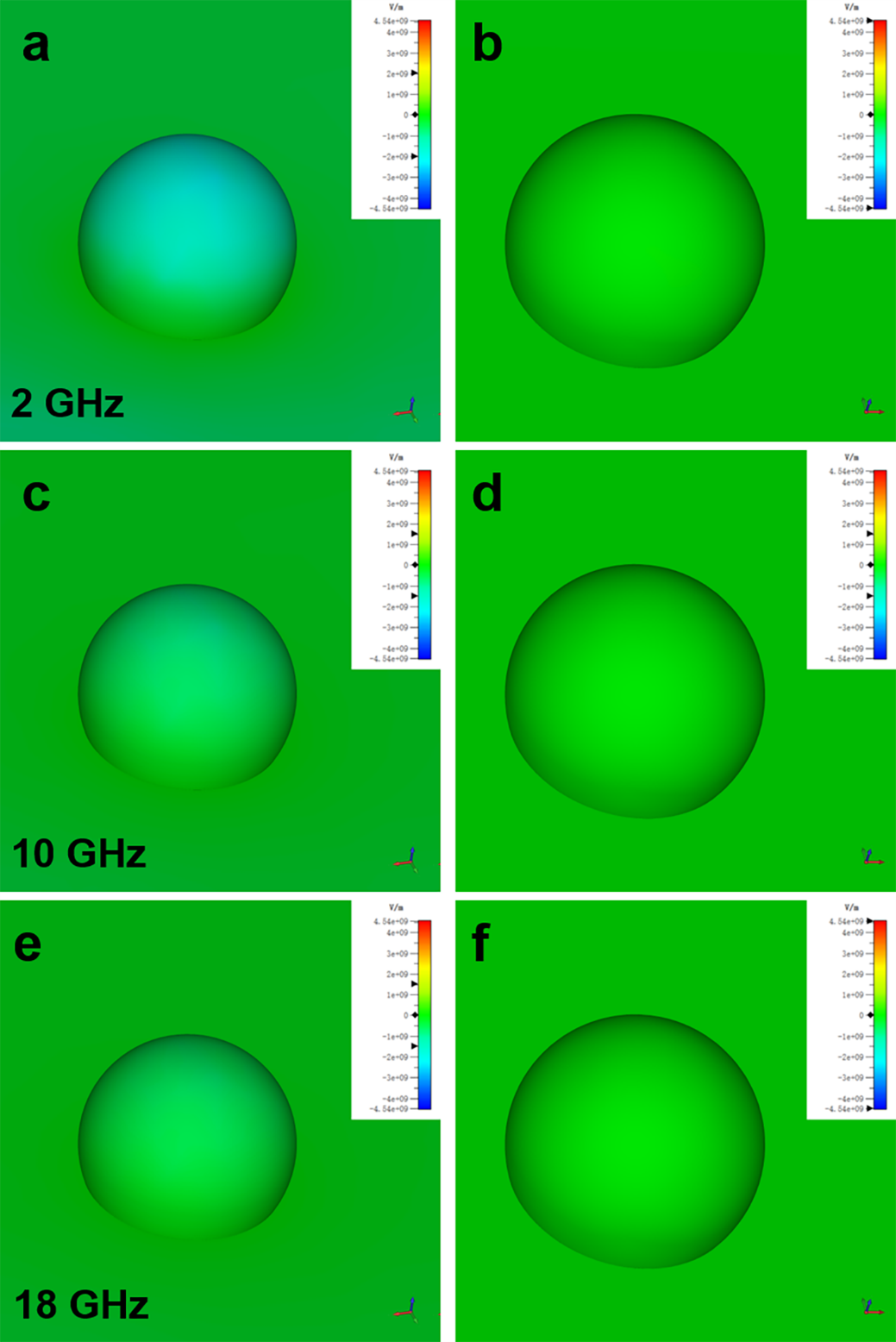


**Figure S28.** Local electric-field distributions at the (a,c,e) top and (b,d,f) middle positions of CoAl/Fe_0.8_Co_0.2_S at frequencies of (a,b) 2 GHz, (c,d) 10 GHz, and (e,f) 18 GHz.


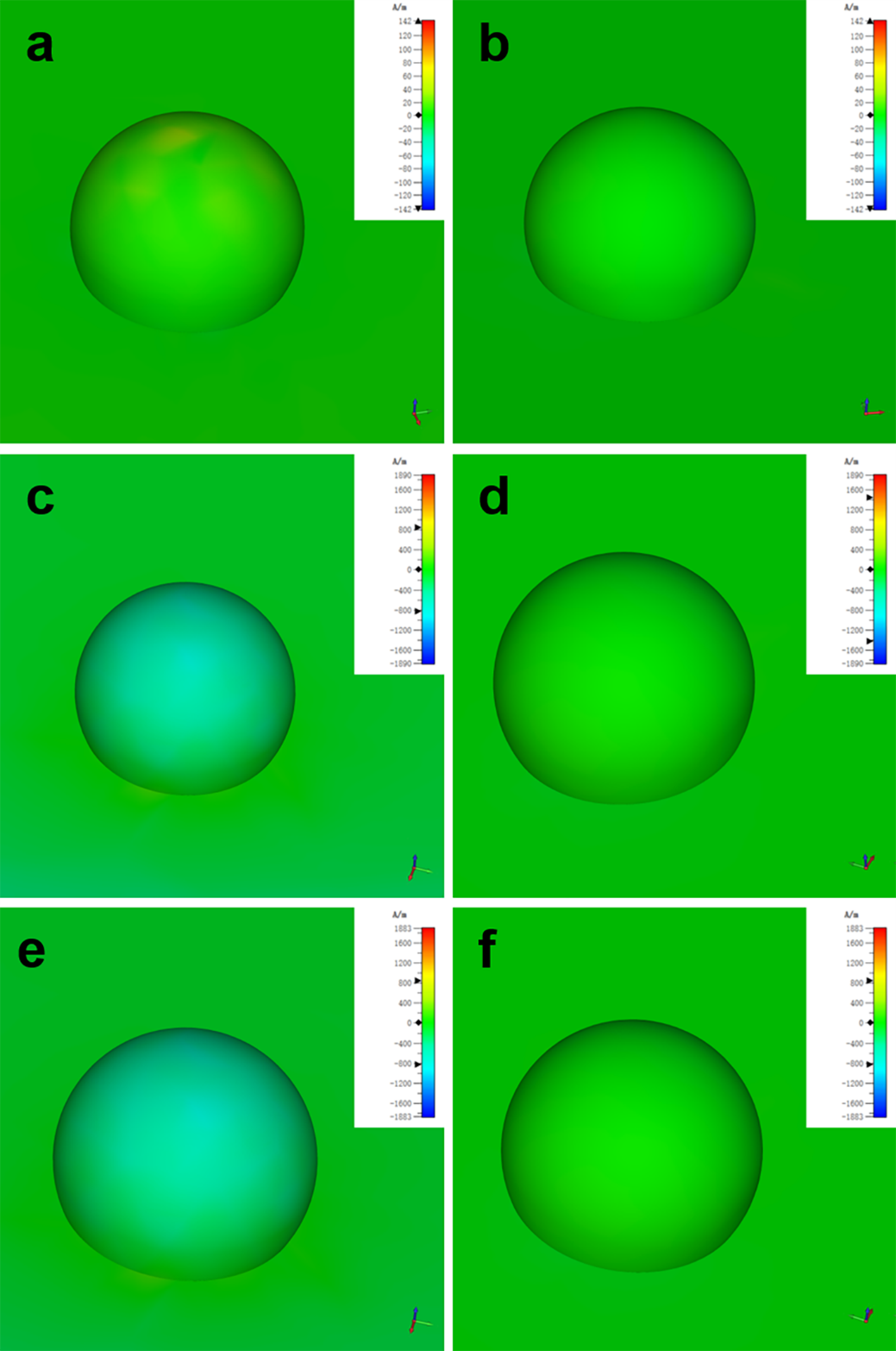


**Figure S29.** Magnetic-field distributions at the (a,c,e) right and (b,d,f) middle positions of (a,b) CoAl/Co_1-x_S, (c,d) CoAl/FeS, and (e,f) CoAl/Fe_0.8_Co_0.2_S at frequency of 2 GHz.


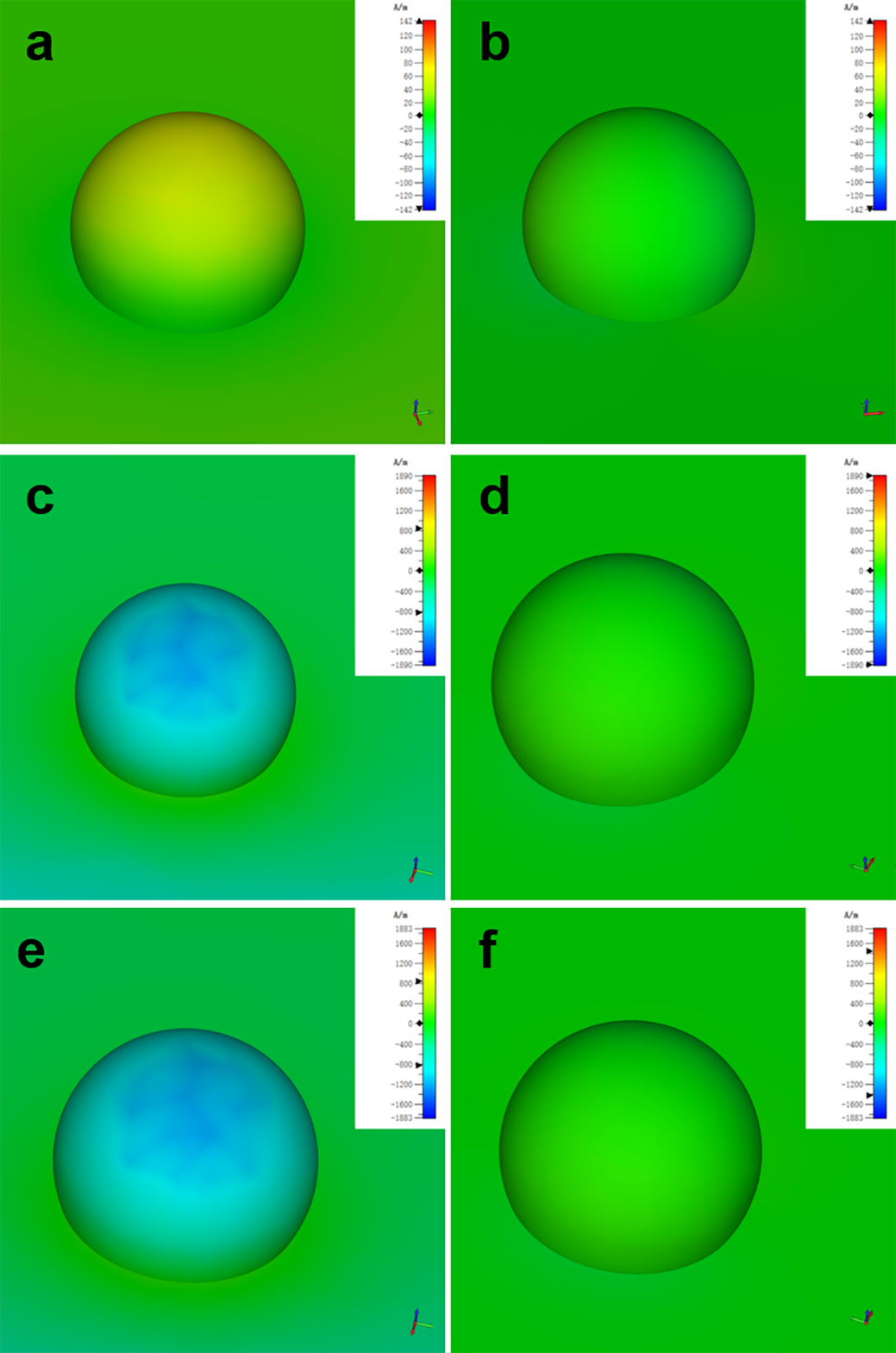


**Figure S30.** Magnetic-field distributions at the (a,c,e) right and (b,d,f) middle positions of (a,b) CoAl/Co_1-x_S, (c,d) CoAl/FeS, and (e,f) CoAl/Fe_0.8_Co_0.2_S at frequency of 10 GHz.


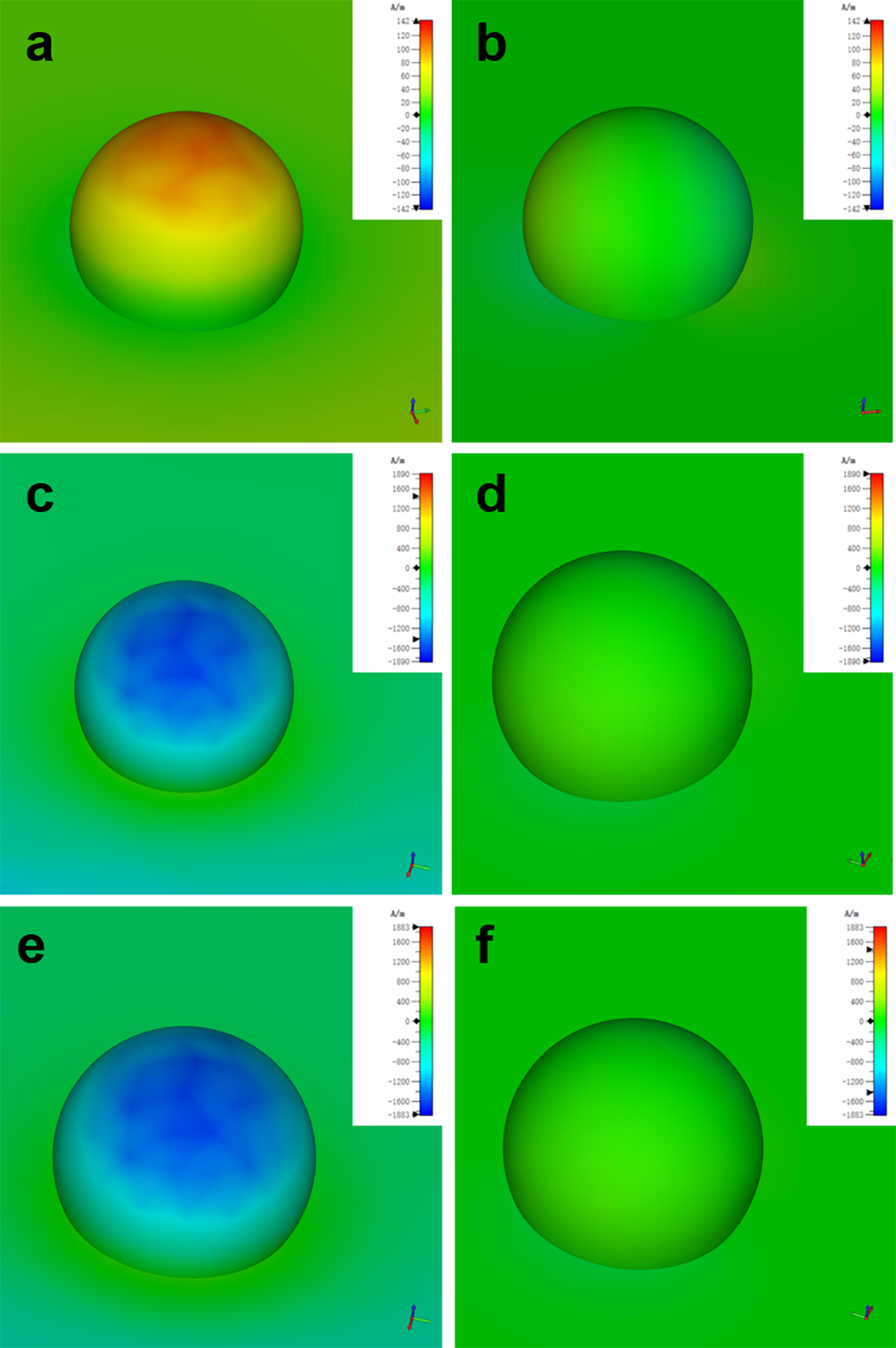


**Figure S31.** Magnetic-field distributions at the (a,c,e) right and (b,d,f) middle positions of (a,b) CoAl/Co_1-x_S, (c,d) CoAl/FeS, and (e,f) CoAl/Fe_0.8_Co_0.2_S at frequency of 18 GHz.

**Table S1.** EMW absorption performance of CoAl/Fe_0.8_Co_0.2_S and recently advanced metal sulfide-based absorbers.

| Samples | Materials | Shapes | *d*  /mm | *R*_L_  /dB | *R*_L_/*d*  /dB mm^-1^ | Frequency  /GHz | Bandwidth (< -10 dB)  /GHz | Ref. |
| --- | --- | --- | --- | --- | --- | --- | --- | --- |
| 1 | Fe_3_O_4_/FeS_2_/Fe_7_S_8_ | Nanoflowers | 5.00 | -23.78 | -4.76 | 5.10 | 2.40 | [5] |
| 2 | CoFe_2_O_4_@1T/2H-MoS_2_ | Nanoflowers | 4.50 | -28.00 | -6.22 | 5.00 | 2.00 | [6] |
| 3 | MXene/NiCo_2_S_4_ | Nanoflowers | 5.00 | -28.00 | -5.60 | 5.40 | 1.40 | [7] |
| 4 | NiS_2_/SnS_2_ | Nanoflowers | 4.00 | -30.00 | -7.50 | 4.95 | 2.00 | [8] |
| 5 | CoS_2_@MoS_2_/rGO | Nanoflowers | 4.00 | -30.00 | -7.50 | 6.00 | 0.90 | [9] |
| 6 | CoSe_2-x_S | Nanospheres | 4.00 | -31.50 | -7.88 | 5.60 | 1.50 | [10] |
| 7 | CC@NPC/CoS_2_ | Nanowires | 3.50 | -32.00 | -9.14 | 5.80 | 1.00 | [11] |
| 8 | Fe_3_O_4_/FeS_2_/Fe_7_S_8_ | Nanosheets | 4.50 | -32.00 | -7.11 | 4.20 | 2.60 | [12] |
| 9 | Ni_x_S_y_ | Nanowires | 3.50 | -32.00 | -9.14 | 5.90 | 1.00 | [13] |
| 10 | MWCNTs/CoS | Nanoflowers | 4.50 | -33.00 | -7.33 | 5.40 | 1.50 | [14] |
| 11 | Zn_0.76_Co_0.24_S-Co_3_S_4_ | Nanospheres | 3.60 | -33.00 | -9.17 | 6.00 | 1.80 | [15] |
| 12 | MoS_2_/Fe_3_O_4_ | Nanoflowers | 4.00 | -33.50 | -8.38 | 5.10 | 1.40 | [16] |
| 13 | Fe_3_O_4_@1T/2H-MoS_2_ | Nanoflowers | 9.07 | -33.50 | -3.69 | 2.80 | 1.30 | [17] |
| 14 | PC@CuS | Nanoflowers | 4.00 | -34.00 | -8.50 | 5.80 | 1.30 | [18] |
| 15 | Co_x_Fe_3-x_O_4_/MoS_2_ | Nanospheres | 5.00 | -35.00 | -7.00 | 5.10 | 1.80 | [19] |
| 16 | Co_9_S_8_@C | Nanospheres | 5.00 | -36.00 | -7.20 | 4.20 | 1.00 | [20] |
| 17 | Ti_3_C_2_T_x_/Co_9_S_8_ | Nanosheets | 4.60 | -36.42 | -7.92 | 4.95 | 1.00 | [21] |
| 18 | Fe_3_O_4_@SiO_2_@MoS_2_ | Nanospheres | 4.44 | -37.00 | -8.33 | 5.10 | 1.80 | [22] |
| 19 | MoO_3_/MoS_2_ | Nanowires | 3.50 | -37.00 | -10.57 | 5.00 | 1.20 | [23] |
| 20 | Fe_3_O_4_/Fe_3_S_4_ | Nanosheets | 5.50 | -37.30 | -6.78 | 5.40 | 2.00 | [24] |
| 21 | MoS_2_-CNTs | Nanowires | 5.00 | -37.50 | -7.50 | 5.00 | 1.60 | [25] |
| 22 | PPy@MoS_2_ | Flakes | 5.50 | -37.70 | -6.85 | 5.60 | 1.30 | [26] |
| 23 | CoZn/C@MoS_2_ | Nanosheets | 4.50 | -41.50 | -9.22 | 5.36 | 1.50 | [27] |
| 24 | Fe_3_O_4_/Fe_7_S_8_@C | Nanoflowers | 4.00 | -41.50 | -10.38 | 5.50 | 1.50 | [28] |
| 25 | MoS_2_@Gd_2_O_3_ | Nanospheres | 4.00 | -43.50 | -10.88 | 5.80 | 2.20 | [29] |
| 26 | CF-Mil88A@MoS_2_ | Nanowires | 3.70 | -44.50 | -12.03 | 5.10 | 1.80 | [30] |
| 27 | Co_1-x_S/Co_9_S_8_ | Nanospheres | 4.00 | -50.00 | -12.50 | 4.20 | 1.30 | [31] |
| 28 | Co-C/Co_9_S_8_ | Nanospheres | 4.50 | -51.68 | -11.48 | 5.20 | 1.80 | [32] |
| 29 | Co_0.6_Fe_2.4_O_4_@MoS_2_ | Nanoflowers | 7.22 | -52.50 | -7.27 | 3.00 | 1.50 | [33] |
| 30 | Aerogel@Co_9_S_8_ | Nanoparticles | 5.20 | -53.60 | -10.31 | 3.00 | 1.00 | [34] |
| 31 | Co-C/Co_9_S_8_ | Nanospheres | 4.89 | -54.02 | -11.05 | 3.04 | 1.00 | [35] |
| 32 | CNTs@MoS_2_ | Nanowires | 4.57 | -56.53 | -12.37 | 5.50 | 1.10 | [36] |
| 33 | MnS@MoS2 | Nanoflowers | 5.47 | -56.78 | -10.38 | 5.28 | 2.00 | [37] |
| 34 | VS_2_ | Nanosheets | 5.75 | -57.50 | -10.00 | 4.00 | 1.50 | [38] |
| 35 | MoS_2_@HCS | Nanospheres | 5.00 | -58.00 | -11.60 | 6.00 | 1.20 | [39] |
| - | CoAl/Fe_0.8_Co_0.2_S | Nanosheets | 3.50 | -65.26 | -18.65 | 6.00 | 0.80 | This work |

Note: The exact *R*_L_ values, thickness, and bandwidth were not presented in some references, thus, those values were dug out according to the *R*_L_-f curves in the C band.

**References**

[1] Ou, B.; Wang, J.; Wu, Y.; Zhao, S.; Wang, Z. Efficient removal of Cr (VI) by magnetic and recyclable calcined CoFe-LDH/g-C_3_N_4_ via the synergy of adsorption and photocatalysis under visible light. *Chem. Eng. J.* **2020**, *380*, 122600.

[2] Huang, X.; Yu, G.; Zhang, Y.; Zhang, M.; Shao, G. Design of cellular structure of graphene aerogels for electromagnetic wave absorption. *Chem. Eng. J.* **2021**, *426*, 13189.

[3] Wang, J.; Zhou, M.; Xie, Z.; Hao, X.; Tang, S.; Wang, J.; Zou, Z.; Ji, G. Enhanced interfacial polarization of biomass-derived porous carbon with a low radar cross-section. *J. Colloid and Interf. Sci.* **2022**, *612*, 146-155.

[4] Zou, Z.; Ning, M.; Lei, Z.; Zhuang, X.; Tan, G.; Hou, J.; Xu, H.; Man, Q.; Li, J.; Li, R. 0D/1D/2D architectural Co@C/MXene composite for boosting microwave attenuation performance in 2-18 GHz. *Carbon* **2022**, *193*, 182-194.

[5] Liu, J.; Wang, M.; Zhang, L.; Zang, D.; Liu, H.; Liotta, L.; Wu, H. Tunable sulfur vacancies and hetero-interfaces of FeS_2_-based composites for high-efficiency electromagnetic wave absorption. *J. Colloid Interf. Sci.* **2021**, *591*, 148-160.

[6] Wang, X.; Zhu, T.; Chang, S.; Lu, Y.; Mi, W.; Wang, W. 3D Nest-Like Architecture of Core-Shell CoFe_2_O_4_@1T/2HMoS_2_ Composites with Tunable Microwave Absorption Performance. *ACS Appl. Mater. Interfaces* **2020**, *12*, 11252-11264.

[7] Li, X.; Yin, S.; Cai, L.; Wang, Z.; Zeng, C.; Jiang, H.; Cheng, J.; Lu, W. Sea-urchin-like NiCo_2_S_4_ modified MXene hybrids with enhanced microwave absorption performance. *Chem. Eng. J.* **2023**, *454*, 140127.

[8] Dong, Y.; Zhu, X.; Pan, F.; Xiang, Z.; Zhang, X.; Cai, L.; Lu, W. Fire-retardant and thermal insulating honeycomb-like NiS_2_/SnS_2_ nanosheets@3D porous carbon hybrids for high-efficiency electromagnetic wave absorption. *Chem. Eng. J.* **2021**, *426*, 131272.

[9] Zhu, T.; Shen, W.; Wang, X.; Song, Y.; Wang, W. Paramagnetic CoS_2_@MoS_2_ core-shell composites coated by reduced graphene oxide as broadband and tunable high-performance microwave absorbers. *Chem. Eng. J.* **2018**, *19*, 31553.

[10] Liu, J.; Zhang, L.; Wu, H. Anion-Doping-Induced Vacancy Engineering of Cobalt Sulfoselenide for Boosting Electromagnetic Wave Absorption. *Adv. Funct. Mater.* **2022**, *32*, 2200544.

[11] Liu, P.; Zhu, C.; Gao, S.; Guan, C.; Huang, Y.; He, W. N-doped porous carbon nanoplates embedded with CoS_2_ vertically anchored on carbon cloths for flexible and ultrahigh microwave absorption. *Carbon* **2020**, *163*, 348-359.

[12] Wu, H.; Liu, J.; Liang, H.; Zang, D. Sandwich-like Fe_3_O_4_/Fe_3_S_4_ composites for electromagnetic wave absorption. *Chem. Eng. J*. **2020**, *393*, 124743.

[13] Zhu, Q.; Zhang, X.; Zheng, Y.; Xia, Y.; Nie, Z.; Zhang, W.; Yan, H.; Qi, S. Co_x_S_y_/C@MoS_2_ nanofibers: synthesis, characterization and microwave absorption investigation. *J. Mater. Sci.: Mater. Electron*. **2020**, *32*, 25782-25794.

[14] Huang, T.; He, M.; Zhou, Y.; Pan, W.; Li, S.; Ding, B.; Huang, S.; Tong, Y. Fabrication and microwave absorption of multiwalled carbon nanotubes anchored with CoS nanoplates. *J. Mater. Sci.: Mater. Electron*. **2017**, *28*, 7622-7632.

[15] Liu, J.; Zhang, L.; Wu, H.; Zang, D. Boosted electromagnetic wave absorption performance from vacancies, defects and interfaces engineering in Co(OH)F/Zn_0.76_Co_0.24_S/ Co_3_S_4_ composite. *Chem. Eng. J*. **2021**, *411*, 128601.

[16] Zhang, D.; Chai, J.; Cheng, J.; Jia, Y.; Yang, X.; Wang, H.; Zhao, Z.; Han, C.; Shan, G.; Zhang, W.; Zheng, G.; Cao, M. Highly Efficient Microwave Absorption Properties and Broadened Absorption Bandwidth of MoS_2_-iron Oxide Hybrids and MoS_2_-based Reduced Graphene Oxide Hybrids with Hetero-structures. *Appl. Surf. Sci*. **2018**, *462*, 872-882.

[17] Wu, M.; Liang, X.; Zheng, Y.; Qian, C.; Wang, D. Excellent microwave absorption performances achieved by optimizing core@shell structures of Fe_3_O_4_@1T/2H-MoS_2_ composites. *J. Alloy Compd*. **2022**, *910*, 164881.

[18] Zhang, X.; Cai, L.; Xiang, Z.; Lu, W. Hollow CuS microﬂowers anchored porous carbon composites as lightweight and broadband microwave absorber with ﬂame-retardant and thermal stealth functions. *Carbon* **2021**, *184*, 514-525.

[19] Long, L.; Yang, E.; Qi, X.; Xie, R.; Bai, Z.; Qin, S.; Deng, G.; Zhong, W. Positive and Reverse Core/Shell Structure Co_x_Fe_3-x_O_4_/MoS_2_ and MoS_2_/Co_x_Fe_3-x_O_4_ Nanocomposites: Selective Production and Outstanding Electromagnetic Absorption Comprehensive Performance. *ACS Sustainable Chem. Eng*. **2020,** *8*, 613-623.

[20] Liang, N.; Yin, Z.; Guo, J.; Fang, W.; Wang, Q.; Tian, G.; Zhang, D.; Yue, H.; Feng, S. Yolk-shell structure synergistic defect engineering for boosting electromagnetic wave absorption in Co_9_S_8_@Humins-derived carbon. [*J. Mater. Sci. Technol*](https://www.baidu.com/s?tn=85070231_43_hao_pg&wd=J%20MATER%20SCI%20TECHNOL&usm=3&ie=utf-8&rsv_pq=b78bfed6001925f4&oq=Journal%20of%20Materials%20Science%20%26%20Technology%E7%BC%A9%E5%86%99&rsv_t=8408LTBWcCinLnPchVR%2BjKAZ5ZBXTNH21%2FHzgSUo6XwoWC%2BJiOt216rxVKAPv%2FZkPKAuURFch1it&sa=re_dqa_zy&icon=1). **2023**, *164*, 140-149.

[21] Hou, T.; Jia, Z.; Wang, B.; Li, H.; Liu, X.; Bi, L. MXene-based accordion 2D hybrid structure with Co_9_S_8_/C/Ti_3_C_2_T_x_ as efficient electromagnetic wave absorber. *Chem. Eng. J.* **2021**, *414*, 128875.

[22] Liao, J.; Qiu, J.; Wang, G.; Du, R.; Tsidaeva, N.; Wang, W. 3D core-shell Fe_3_O_4_@SiO_2_@MoS_2_ composites with enhanced microwave absorption performance. *J. Colloid Interf. Sci.* **2021**, *604*, 537-549.

[23] Li, C.; Shen, X.; Ding, R.; Wang, G. Controllable Synthesis of One-dimensional MoO_3_/MoS_2_ Hybrid Composites with their Enhanced Efficient Electromagnetic Wave Absorption Properties. *ChemPlusChem* **2018**, *84*, 226-232.

[24] Cheng, J.; Zhang, H.; Wang, H.; Huang, Z.; Raza, H.; Hou, C.; Zheng, G.; Zhang, D.; Zheng, Q.; Che, R. Tailoring Self-Polarization of Bimetallic Organic Frameworks with Multiple Polar Units Toward HighPerformance Consecutive Multi-Band Electromagnetic Wave Absorption at Gigahertz. *Adv. Funct. Mater*. **2022**, *32*, 2201129.

[25] Sun, Y.; Zhong, W.; Wang, Y.; Xu, X.; Wang, T.; Wu, L.; Du, Y. MoS_2_ Based Mixed-dimensional van der Waals Heterostructures: a New Platform for Excellent and Controllable Microwave Absorption Performance. *ACS Appl. Mater. Interfaces* **2017**, *14*, 34243-34255.

[26] Gai, L.; Zhao, Y.; Song, G.; An, Q.; Xiao, Z.; Zhai, S.; Li, Z. Construction of core-shell PPy@MoS_2_ with nanotube-like heterostructures for electromagnetic wave absorption: Assembly and enhanced mechanism. *Compos. Part A* **2020**, *136*, 105965.

[27] Bi, Y.; Ma, M.; Liu, Y.; Tong, Z.; Wang, R.; Chung, K.; Ma, A.; Wu, G.; Ma, Y.; He, C.; Liu, P.; Hu, L. Microwave absorption enhancement of 2-dimensional CoZn/C@MoS_2_@PPy composites derived from metal-organic framework. *J. Colloid Interf. Sci*. **2021**, *600*, 209-218.

[28] Wang, Y.; Cheng, R.; Cui, W.; Lu, Z.; Yang, X.; Pan, H.; Che, R. Heterostructure design of 3D hydrangea-like Fe_3_O_4_/Fe_7_S_8_@C core-shell composite as a high-efficiency microwave absorber. *Carbon* **2023**, *210*, 118043.

[29] Shen M.; Qi J.; Xu X.; Li J.; Xu Y.; Yang H.; Gao K.; Huang J.; Li J.; Shang Z.; Ni Y. Promoting electromagnetic wave absorption performance by integrating MoS_2_@Gd_2_O_3_/MXene multiple hetero-interfaces in wood-derived carbon aerogels. *Small* **2024**, *20*, 2306915.

[30] Zhao, X.; Huang, Y.; Liu, X.; Yu, M.; Zong, M.; Li, T. Magnetic nanorods/carbon fibers heterostructures coated with flower-like MoS_2_ layers for superior microwave absorption. *Carbon* **2023**, *213*, 118265.

[31] Zhang, Y.; Zhang, Y.; Yu, J.; Miao, X.; Li, Y.; Xu, F. Hollow Co_9_S_8_ nanoparticles with carbon shells for wide-band and efficient microwave absorption. *J. Alloy Compd*. **2022**, *926*, 166875.

[32] Li, Y.; Gai, L.; Song, J.; An, Q.; Xiao, Z.; Zhai, S. Enhanced properties of CoS_2_/Cu_2_S embedded N/S co-doped mesh-like carbonaceous composites for electromagnetic wave absorption. *Carbon* **2022**, *186*, 238-252.

[33] Long, L.; Yang, E.; Qi, X.; Xie, R.; Bai, Z.; Qin, S.; Zhong, W. Core@shell structured flower-like Co_0.6_Fe_2.4_O_4_@MoS_2_ nanocomposites: a strong absorption and broadband electromagnetic wave absorber. *J. Mater. Chem. C* **2019**, *7*, 8975-8981.

[34] Cui, C.; Geng, L.; Jiang, S.; Bai, W.; Dai, L.; Jiang, S.; Hu, J.; Ren, E.; Guo, R. Construction of hierarchical carbon fiber aerogel@hollow Co_9_S_8_ polyhedron for high-performance electromagnetic wave absorption at low-frequency. *Chem. Eng. J.* **2023**, *466*, 143122.

[35] Liu, X.; Hao, C.; He, L.; Yang, C.; Chen, Y.; Jiang, C.; Yu, R. Yolk-shell structured Co-C/Void/Co_9_S_8_ composites with a tunable cavity for ultrabroadband and efficient lowfrequency microwave absorption. *Nano Res*. **2018**, *11*, 5974.

[36] Wang, R.; Yang, E.; Qi, X.; Xie, R.; Qin, S.; Deng, C.; Zhong, W. Constructing and optimizing core@shell structure CNTs@MoS_2_ nanocomposites as outstanding microwave absorbers. *Appl. Surf. Sci.* **2020**, *516*, 146159.

[37] Tian, X.; Wang, Y.; Wang, J.; Guo, Z.; Hu, L.; Tang, C.; Chen, G.; Fang, Y. One-Pot Hydrothermal Synthesis of Core-Shell MnS@MoS_2_ Heterojunction for Enhanced Microwave Absorption. *Adv. Eng. Mater*. **2023**, *25*, 2201526.

[38] Zhang, X.; Cai, L.; Xiang, Z.; Lu, W. Hollow CuS microﬂowers anchored porous carbon composites as lightweight and broadband microwave absorber with ﬂame-retardant and thermal stealth functions. *Carbon* **2021**, *184*, 514-525.

[39] Ning, M.; Man, Q.; Tan, G.; Lei, Z.; Li, J.; Li, R. Ultrathin MoS_2_ Nanosheets Encapsulated in Hollow Carbon Sphere: A case of Dielectric Absorber with optimized Impedance for Efficient Microwave Absorption. *ACS Appl. Mater. Interfaces* **2020**, *12*, 20785-20796.
